# PthA4^AT^, a 7.5‐repeats transcription activator‐like (TAL) effector from *Xanthomonas citri* ssp. *citri*, triggers citrus canker resistance

**DOI:** 10.1111/mpp.12844

**Published:** 2019-07-05

**Authors:** Roxana Andrea Roeschlin, Facundo Uviedo, Lucila García, María Celeste Molina, María Alejandra Favaro, María Amalia Chiesa, Sabrina Tasselli, José Manuel Franco‐Zorrilla, Javier Forment, José Gadea, María Rosa Marano

**Affiliations:** ^1^ Instituto de Biología Molecular y Celular de Rosario (IBR)‐Consejo Nacional de Investigaciones Científicas y Tecnológicas (CONICET) Ocampo y Esmeralda S/N S2002LRK Rosario Argentina; ^2^ Área Virología, Facultad de Ciencias Bioquímicas y Farmacéuticas Universidad Nacional de Rosario (UNR) Suipacha 590 S2002LRK Rosario Argentina; ^3^ Unidad Genómica, Centro Nacional de Biotecnología (CNB)‐Consejo Superior de Investigaciones Científicas (CSIC) Darwin 3 28049 Madrid España; ^4^ Instituto de Biología Molecular y Celular de Plantas (IBMCP) Universidad Politécnica de Valencia‐CSIC Ingeniero Fausto Elio S/N. 46022 Valencia España; ^5^ Present address: Facultad de Ciencias Agropecuarias Universidad Católica de Santa Fe Ludueña 612 S3560DYR Santa Fe Argentina; ^6^ Present address: Facultad de Ciencias Agrarias Universidad Nacional del Litoral, Producción Vegetal Kreder 2805, 3080 HOF Esperanza Santa Fe Argentina; ^7^ Present address: Laboratorio de Fisiología Vegetal Instituto de Investigaciones en Ciencias Agrarias de Rosario (IICAR)‐UNR/CONICET Parque Villarino S/N 2125 Zavalla, Santa Fe Argentina

**Keywords:** citrus, hypersensitive response (HR), *Nicotiana benthamiana*, transcription activator‐like (TAL) effectors, *Xanthomonas citri*

## Abstract

Transcription activator‐like effectors (TALEs) are important effectors of *Xanthomonas* spp. that manipulate the transcriptome of the host plant, conferring susceptibility or resistance to bacterial infection. *Xanthomonas citri* ssp. *citri* variant A^T^ (*X. citri* A^T^) triggers a host‐specific hypersensitive response (HR) that suppresses citrus canker development. However, the bacterial effector that elicits this process is unknown. In this study, we show that a 7.5‐repeat TALE is responsible for triggering the HR. PthA4^AT^ was identified within the *pthA* repertoire of *X. citri* A^T^ followed by assay of the effects on different hosts. The mode of action of PthA4^AT^ was characterized using protein‐binding microarrays and testing the effects of deletion of the nuclear localization signals and activation domain on plant responses. PthA4^AT^ is able to bind DNA and activate transcription in an effector binding element‐dependent manner. Moreover, HR requires PthA4^AT^ nuclear localization, suggesting the activation of executor resistance (*R*) genes in host and non‐host plants. This is the first case where a TALE of unusually short length performs a biological function by means of its repeat domain, indicating that the action of these effectors to reprogramme the host transcriptome following nuclear localization is not limited to ‘classical’ TALEs.

## Introduction

Understanding how plants perceive and hamper the attack by potential pathogens has been a fundamental question for plant biologists in recent decades. We now know that bacterial infection normally fails because plants carry genes whose products detect conserved pathogen‐associated molecular patterns (PAMPs) and trigger a complex defence response (PAMP‐triggered immunity, PTI) that restricts pathogen growth (Macho and Zipfel, 2014). Evolution has provided many bacteria with the ability to suppress this basal plant immune system and hence facilitate infection through the action of a battery of proteins called effectors (Macho, 2016). These effectors are expressed during the course of the infection and are translocated into the host mesophyll cells by means of the type III secretion system (T3SS) where they interact with specific plant components. As a countermeasure, some plants have evolved the ability to detect specific bacterial effectors, restricting effector‐mediated bacterial infection by triggering a second layer of defence responses called effector‐triggered immunity (ETI) (Toruño *et al*., 2016). The ability of the pathogen to avoid plant recognition relies then on effector evolution, in a never‐ending struggle between both organisms (Carella *et al*., 2018).

Transcription activator‐like effectors (TALEs), belonging to the *Xanthomonas* AvrBs3/PthA family of T3SS effectors, function as eukaryotic transcription factors in plant cells, playing a central role in promoting bacterial disease by the induction of host susceptibility (*S*) genes. Contrary to the mode of action of other families of bacterial effectors that exert their function in the cytoplasm, TALEs are translocated into the plant cell nucleus and bind to the promoter regions of plant target genes whose transcription facilitates bacterial colonization and spread (Boch and Bonas, 2010; Bogdanove *et al*., 2010). The specificity of action of different TALEs is determined by the structure of these proteins. They contain T3SS translocation signals in the N‐terminal region, a central DNA‐binding domain and a C‐terminal region with nuclear localization signals (NLS) followed by an acidic activation domain (AD). TALEs vary mostly in their central region, which consists of a number of tandemly arranged repeats of 33–34 amino acids with hypervariable di‐amino acids at positions 12 and 13, termed the repeat‐variable diresidue (RVD). Each repeat interacts with a nucleotide in the DNA, and these two residues determine the specificity of each repeat for a particular nucleotide. Thus, the RVD sequence of each TALE defines the effector‐binding element (EBE) in the promoter region of the target genes (Boch *et al*., 2009; Moscou and Bogdanove, 2009). Over the course of evolution, some plants have evaded the activation of susceptibility genes by modification of their promoters (Bogdanove *et al*., 2010; Hutin *et al*., 2015), or co‐opted the TALE mechanism of gene activation for resistance, by means of gene traps (executor resistance (*R*) genes) that contain EBEs in their promoter regions, and whose activation by the corresponding TALE triggers a host resistance response (Römer *et al*., 2007; Zhang *et al*., 2015).

*Xanthomonas citri* ssp. *citri* (*X. citri*) is the causal agent of citrus canker, a serious economic disease that provokes losses worldwide. Infected fruits have decreased commercial quality, compromising their acceptance by most markets (Canteros *et al*., 2017; Ference *et al*., 2018). Outbreaks occur sporadically and copper‐based products and eradication of infected trees are the strategies employed to control the disease so far (Behlau *et al*., 2010; Favaro *et al*., 2017). Canker lesions are characterized by raised spongy eruptions caused by bacterial‐induced cell hypertrophy and hyperplasia in leaves and fruits (Graham *et al*., 2004). The pathogenic reference strain *X. citri* 306 contains four TALE genes (*pthA1*, *pthA2*, *pthA3* and *pthA4* harbouring 16.5, 15.5, 15.5 and 17.5 repeats, respectively) located on plasmids pXAC33 (*pthA1*/*pthA2*) and pXAC64 (*pthA3*/*pthA4*) (da Silva *et al*., 2002). TALE PthA4 promotes the expression of citrus lateral organ boundaries gene (*CsLOB1*), a transcription factor involved in hypertrophy and hyperplasia of the cells (Duan *et al*., 1999; Hu *et al*., 2014; Pereira *et al*., 2014). Moreover, TALE PthA1 and PthA3 also contribute to canker symptoms in a host‐dependent manner (Yukari Abe and Benedetti, 2015).

Several reports suggest specific recognition of effectors by host gene products that render plants resistant to *X. citri*, i.e. interactions in which the effector acts as an avirulence factor (Chen *et al*., 2012; Deng *et al*., 2010; Khalaf *et al*., 2011; Lee *et al*., 2009). Recently, we have isolated a *X. citri* strain (*X. citri* A^T^) that triggers a host‐specific defence response in *Citrus limon* that is associated with the interference of biofilm development and arrest of bacterial growth (Chiesa *et al*., 2013; Roeschlin *et al*., 2017). The occurrence of an oxidative burst, the accumulation of salicylic acid and phenolic compounds, and the hypersensitive response (HR) together suggest that it is an ETI response. However, the avirulence effector that elicits these processes is unknown.

In this study, we demonstrate that the causal agent of the triggering of the defence response to *X. citri* A^T^ strain is a short TALE containing 7.5 repeats. In nature, the number of TALEs in a single bacterial strain is highly variable and the RVDs range from 1.5 to 33.5 (Boch and Bonas, 2010; Cox *et al*., 2017; Denancé *et al*., 2018). However, the optimal length for specificity ranges between 15.5 and 19.5 repeats (Rinaldi *et al*., 2017). The screening of *X. citri* diversity in different citrus‐growing regions has identified TALEs ranking from 6.5 to 29 repeats, which likely contain different RVDs. It is suggested that the diversification is driven by recombination, facilitated by the repetitive structure of the central region, leading to variants differing in the number of repeats (Al‐Saadi *et al*., 2007; Gochez *et al*., 2018; Lee *et al*., 2008; Shiotani *et al*., 2007; Ye *et al*., 2013). It has been demonstrated that TALEs shorter than 6.5 repeats are non‐functional in terms of gene activation; these just could be by‐products of recombination events (Boch *et al*, 2009). It is not known, however, if TALEs of intermediate length are functional for gene activation.

Here we show that this 7.5‐repeat TALE of the *X. citri* A^T^ strain is necessary and sufficient to induce HR in *C. limon* and *C. sinensis*. The protein is able to bind DNA *in vitro* and to activate transcription in an EBE‐dependent manner. The HR is triggered only if the TALE protein reaches the plant cell nucleus, and the resistance requires transcriptional activation, suggesting a classical TALE mode of action via activation of executor gene(s).

## Results

### A new repertoire of *pthA* genes is present in the *X. citri* A^T^ strain

**Figure 1:**
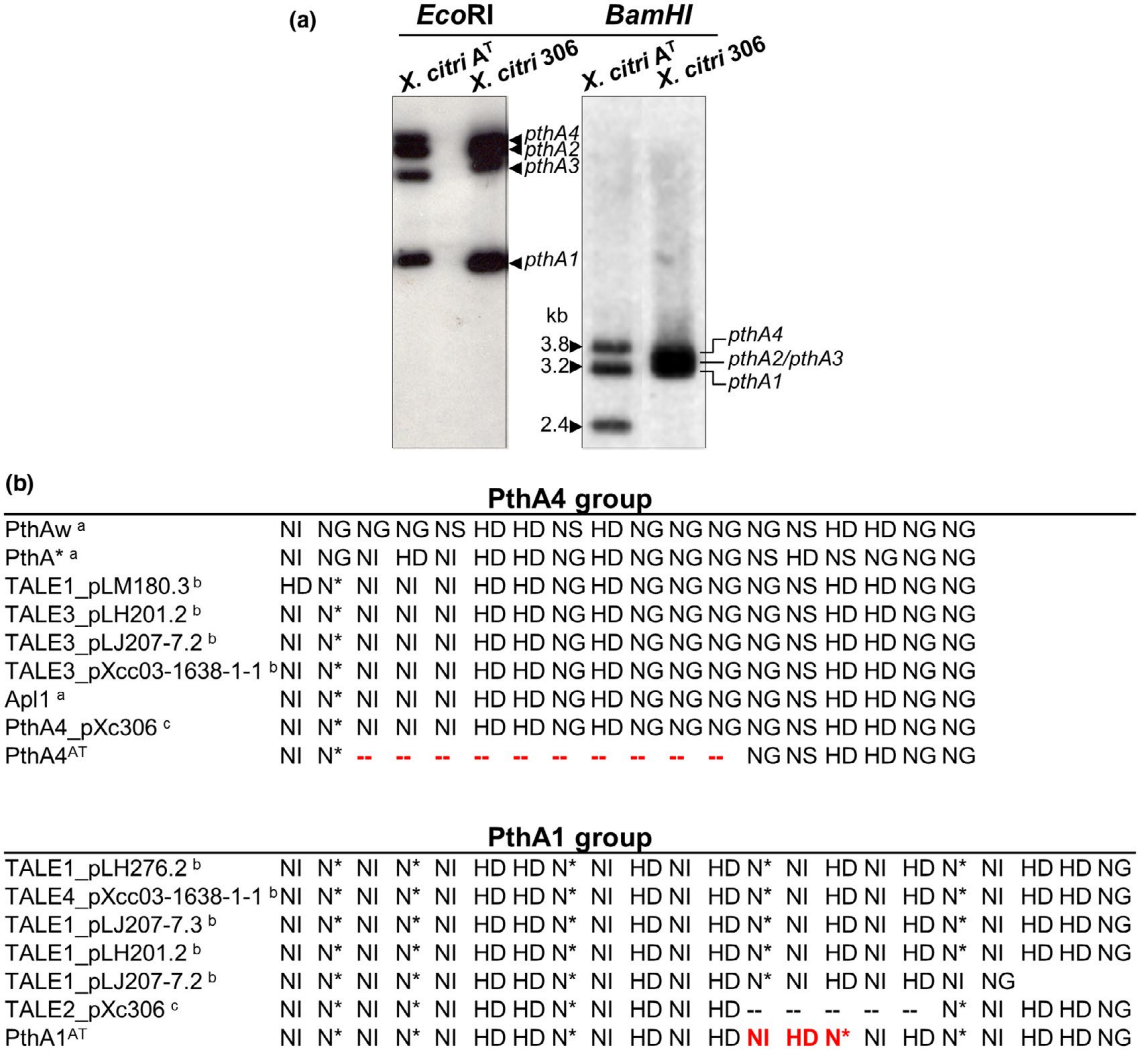
Identification of variants of *pthA* genes in *Xanthomonas citri* A^T^. (a) Southern blot analysis of *X. citri* A^T^ and *X. citri* 306 *pthA* genes. Plasmids were digested with *Eco*RI or *Bam*HI and probed with a *pthA* PCR fragment. Fragments of 3.8, 3.2 and 2.4 kb correspond to *pthA1*, *pthA2/3* and *pthA4* from *X. citri* A^T^, respectively. (b) Alignment of repeat variable di‐residue (RVD) sequences of PthA4 and PthA1. RVD sequences from *X. citri* isolated TALEs were compared with PthA4 and PthA1 from *X. citri* A^T^. Dashed red lines show RVD deleted motif on PthA4^AT^. The residues highlighted in red show the RVDs that differ in positions 13–15 on PthA1^AT^. ^a^Al‐Saadi *et al*. (2007), ^b^Gochez *et al*. (2018), ^c^da Silva *et al*. (2002).

Previous reports have shown a great variability in the size of the *pthA* genes from different *X. citri* strains (Al‐Saadi *et al*., 2007; Gochez *et al*., 2018; Lee *et al*., 2008; Ye *et al*., 2013). Given the mode of action of these TALE proteins, it is expected that this variability will contribute to explain differences in pathogenicity among the strains. To define the *pthA* ‘repertoire’ of the *X. citri* A^T^ strain, Southern blot assays using a *pthA* probe over *Eco*RI‐ and *Bam*HI‐digested DNA of the two *X. citri* plasmids were performed. *Eco*RI digestion of *X. citri* A^T^ showed the presence of four bands, similar to the reference strain *X. citri* 306, indicating the presence of four *pthA* genes. However, after *Bam*HI digestion a new pattern of three discrete 3.8‐, 3.2‐ and 2.4‐kb bands was observed in *X. citri* A^T^ that differed to the one previously reported for *X. citri* 306 (3.4‐kb for *pthA4*, 3.3‐kb for *pthA1* and 3.2‐kb for *pthA2*/*pthA3* genes; da Silva *et al*., 2002) (Fig. 1a). To further investigate the functionality of the *pthA* genes of *X. citri* A^T^, the *Bam*HI bands, revealing the number of repetitions, were cloned and sequenced. The 3.2‐kb band was in fact a double‐band containing the central region of two different *pthA* genes of the same size that were identical to the *pthA2* and *pthA3* genes of *X. citri* 306. However, the two other *pthA* genes differed in the number of central repeats in the coded proteins compared with those of *X. citri* 306. The 3.8‐kb band corresponds to a *pthA* gene encoding a TALE containing 21.5 repeats. The smallest 2.4‐kb *Bam*HI band contains a *pthA* gene encoding a TALE with 7.5 repeat domains. Sequence analysis revealed that the 21.5‐repeat protein is identical to other members of the PthA1 group, differing only in three RVDs in positions 13–15 (Figs 1b and S1). The new 7.5‐repeat protein is identical to PthA4 from *X. citri* 306 in the N‐ and C‐terminal regions, but has an internal deletion of 10 central repeats (Figs 1b and S2). We named these proteins PthA1^AT^ and PthA4^AT^, respectively.

### PthA4^AT^ is necessary and sufficient for the host‐specific induction of hypersensitive response in citrus

Although many genes from *X. citri* 306 contribute to pathogenicity, the *pthA4* 17.5‐repeat TALE gene is a major effector, promoting canker disease symptoms (Duan *et al*., 1999; Da Silva *et al*., 2002; Yukari Abe and Benedetti 2015). To determine if the immune response triggered by *X. citri* A^T^ is still induced in the presence of a full‐length *pthA4* gene, a pBBR plasmid expressing PthA4 from *X. citri* 306 was transferred to *X. citri* A^T^ and the expression was evaluated by western blot (Fig. S3). The *pthA4*–expressing *X. citri* A^T^ was still able to triggered HR in *C. limon* (Fig. S4), indicating that an active PthA4 does not suppress HR.

**Figure 2:**
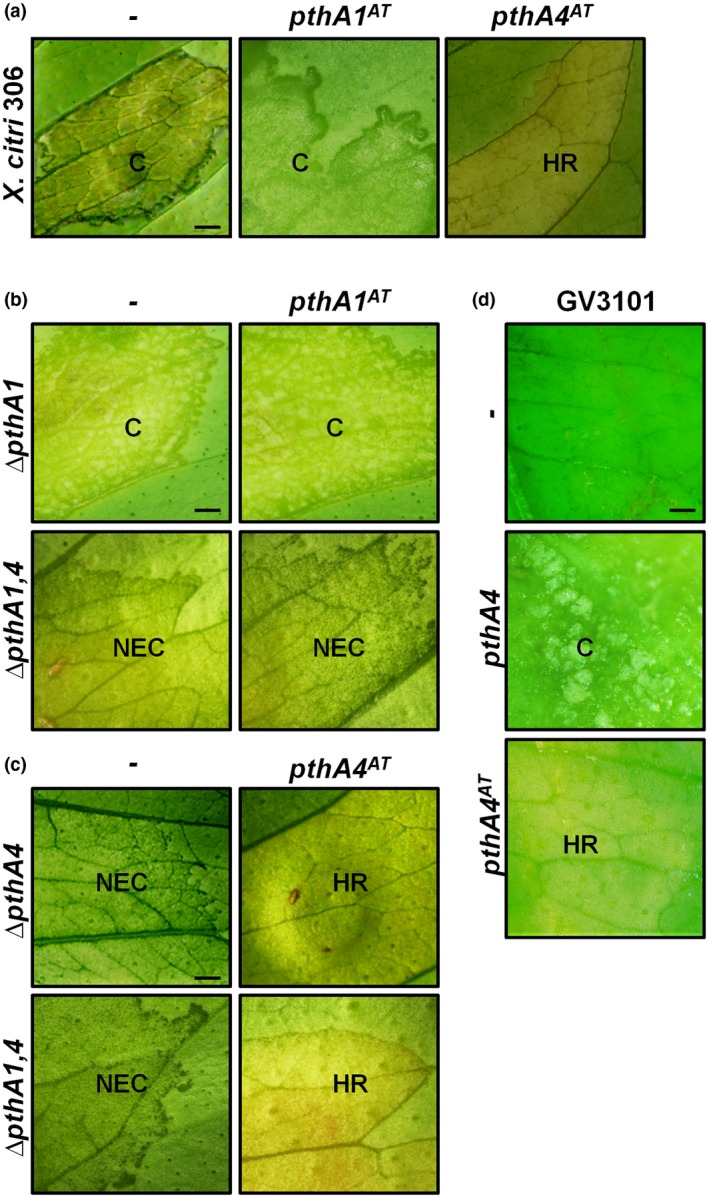
PthA4^AT^ triggers host defence response in *Citrus limon* leaves. (a) Symptoms induced by PthA1^AT^ and PthA4^AT^ on *Xanthomonas citri* 306. (b) and (c) Complementation of *X. citri* mutant strains (Δ*pthA1*, Δ*pthA1,4* and Δ*pthA4*) with PthA1^AT^ or PthA4^AT^. Leaves were infiltrated with the corresponding bacterial suspension and photographed at 15 days post‐inoculation (dpi). (d) *Agrobacterium tumefaciens* GV3101‐mediated transient expressions of PthA4^AT^ and PthA4 on *C. limon* leaves. Leaves were agroinfiltrated and symptoms were photographed at 10 dpi. HR, hypersensitive response; C, canker; NEC, non‐eruptive canker. Scale bar: 10 mm.

To evaluate whether any of the two new *pthA* genes found in *X. citri* A^T^ play any role in this HR, the gene for each TALE was cloned in a pBBR plasmid and expressed in *X. citri* 306 (Fig. S3). No difference in symptoms was observed in *C. limon* after infection with the natural pathogenic *X. citri* 306 or the *pthA1^AT^*‐expressing *X. citri* 306. However, *pthA4^AT^*‐expressing *X. citri* 306 was unable to cause canker symptoms and induced an HR on *C. limon* (Fig. 2a). Similar results were obtained when these two *pthA* genes were expressed in the Argentinian pathogenic T strain, which is related to *X. citri* 306 (Fig. S5). To confirm these results, each of three *X. citri* 306 mutant strains (Δ*pthA1,* Δ*pthA4* or Δ*pthA1,4*) were transformed with either *pthA1^AT^* or *pthA4^AT^*‐expressing plasmids (Fig. S3). The *X. citri* 306 mutant strains expressing *pthA1^AT^* exhibited the same symptoms in *C. limon* as the wild‐type 306 strain (Fig. 2b). By contrast, the presence of PthA4^AT^ either in Δ*pthA4* or Δ*pthA1,4* caused an HR similar to that caused by *X. citri* A^T^ (Fig. 2c). These results suggest that the presence of PthA4^AT^ is necessary for the HR observed in *C. limon*. In further experiments, the *pthA4^AT^* gene was cloned under the control of the CaMV 35S promoter and transiently expressed *in planta*. Agroinfiltration assays showed that transient expression of PthA4^AT^ triggered an HR (Fig. 2d), suggesting that the presence of this protein is necessary and sufficient for the induction of this response in *C. limon*.

### Nuclear localization of PthA4^AT^ is needed to trigger host defense response in *Citrus limon*

**Figure 3:**
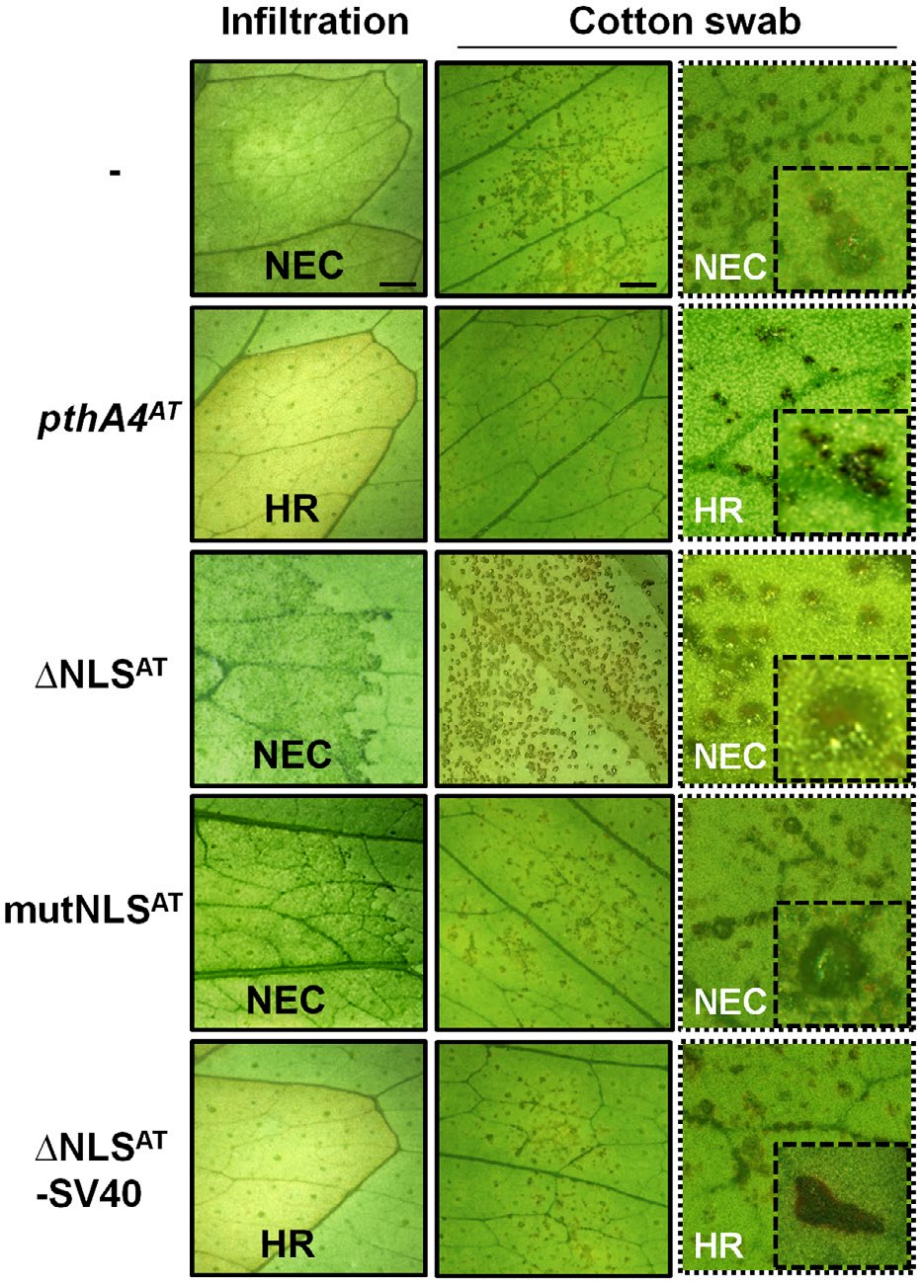
Nuclear localization of PthA4^AT^ is needed to trigger host defence response in *Citrus limon*. Phenotypic response of *Xanthomonas citri* 306 ∆*pthA4* transformed with the different constructs was evaluated in *C. limon* leaves inoculated by pressure infiltration or cotton swab. Photographs were taken 15 days post‐inoculation and the insets show amplification of symptoms. NEC, non‐eruptive canker; HR, hypersensitive response. ∆NLS^AT^:PthA4^AT^ derivative, with an 83‐amino acid deletion in the C‐terminal region, eliminating the three conserved nuclear localization signals (NLS); mutNLS^AT^:PthA4^AT^ derivative mutated in the three NLS; ∆NLS^AT^‐SV40:∆NLS^AT^ derivative containing the NLS of SV40. Scale bar: 10 mm.

The results presented above suggest that PthA4^AT^ recognition by the host plant is the initial step triggering the HR observed in *C. limon*. In order to assess if nuclear localization of PthA4^AT^ is necessary for recognition, two PthA4^AT^‐modified proteins were designed: one with an 87‐amino acid deletion in the C‐terminal region, eliminating the three conserved nuclear localization signals (∆NLS^AT^), and a second one with point mutations in the same three NLS (mutNLS^AT^) (Fig. S6). The constructs were introduced into Δ*pthA4* (Fig. S3) and assayed in *C. limon* following pressure infiltration into the leaves or swab inoculation of the leaf surface. As observed in Fig. 3, none of the two mutant versions of the protein impaired in nuclear localization was able to trigger an HR. Similar results were obtained when *X. citri* 306 was transformed with these constructs (Fig. S7). Moreover, when the NLS of the SV40 (Kalderon *et al*., 1984; Szurek *et al*., 2001) was fused to the impaired ∆NLS^AT^ protein (Fig. S6), the HR was restored (Fig. 3). Taken together, these results indicate that PthA4^AT^ must be localized in the plant cell nucleus to trigger the defence response.

### PthA4^AT^ is able to bind DNA following the TALE code and to activate transcription in an EBE‐dependent manner

**Figure 4:**
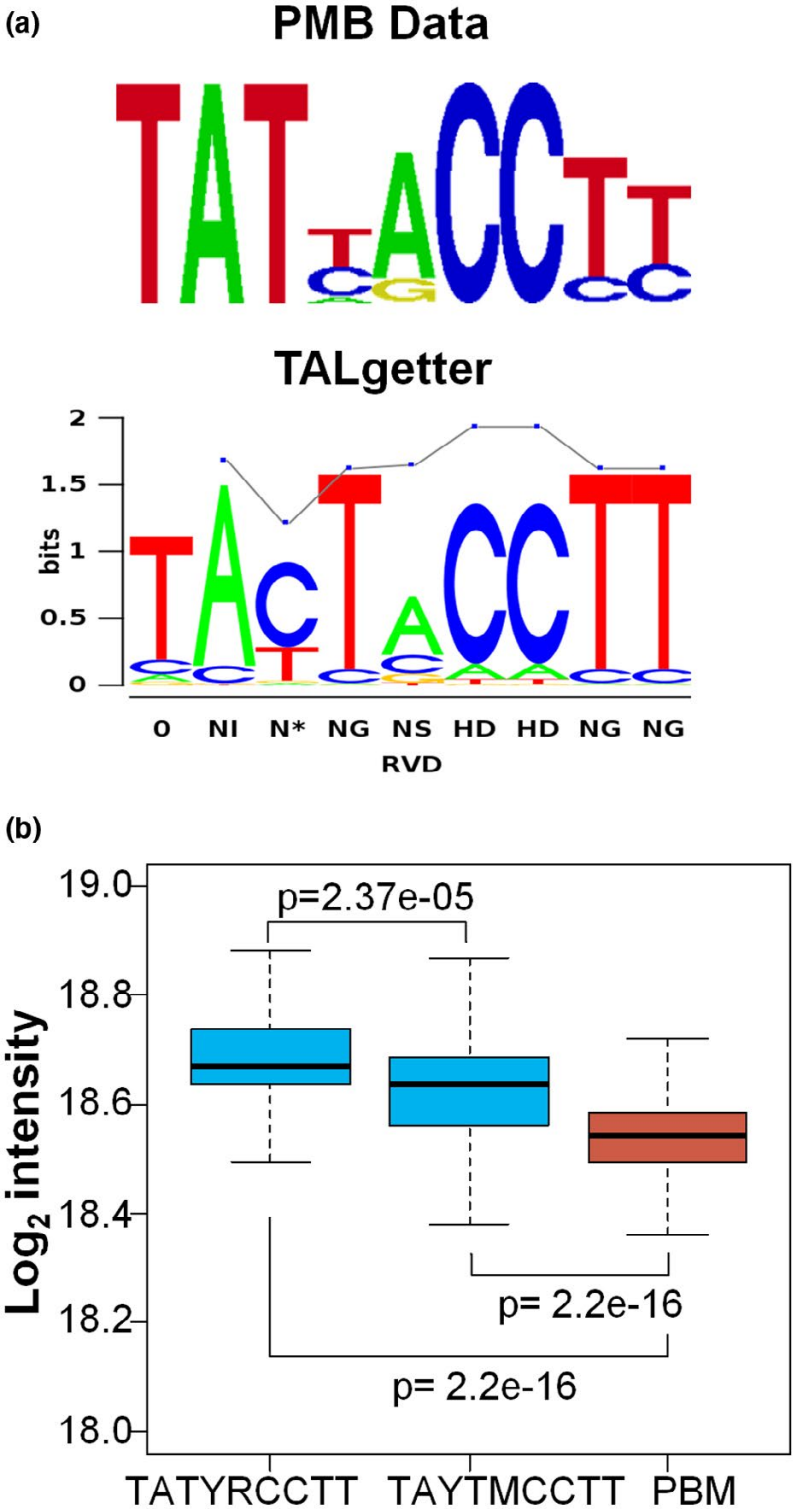
DNA‐binding of PthA4^AT^ follows the TALE code. (a) Consensus predicted sequences for PthA4^AT^ obtained after protein binding microarray (PBM) assay (*E*‐score > 0.45) and TALgetter using *Citrus sinensis* promotorome for the analysis. (b) Relative binding of PthA4^AT^ to different DNA motifs. The box plot represents the distribution of intensities of the DNA probes containing the sequence element bound by PthA4^AT^ with the highest affinity (TATYRCCTT) and the intensities of probes covering the motif predicted by TALgetter (TAYTMCCTT), both in light blue. PthA4^AT^ recognizes both DNA elements, but binding to PBM‐derived motif is higher than to that from TALgetter. Red box corresponds to the distribution of intensities of all the probes in the PBM array. Statistical differences between the different distributions were calculated with the Wilcox exact test. Nucleotide codes are as follows: Y, C or T; M, A or C; R, A or G.

To elucidate whether the 7.5‐repeat PthA4^AT^ TALE was able to bind to DNA *in vitro*, and to determine the specificity of binding, a PBM11 protein‐binding microarray was employed (Godoy *et al*., 2011) that contains all possible double‐stranded 11‐mers (approximately 4.2 million sequences) in approximately 180 000 oligonucleotides of 35 bp. Probing PBM11 with PthA4^AT^ for determination of DNA‐binding specificity yielded an E‐score of 0.48 to the TATTACCTT sequence, with similar affinity to variants of this motif containing one mismatch (Table S6). Considering that it is generally assumed that motifs with *E*‐score > 0.45 reflect high binding specificity (Berger and Bulyk, 2009; Weirauch *et al*., 2013, 2014), we obtained the consensus recognition sequence for PthA4^AT^ from DNA motifs with *E*‐scores > 0.45 (Fig. 4a). This motif matches quite well with the expected binding domain for PthA4^AT^ according to its TALE code (Boch *et al*., 2009).

*X. citri* A^T^ generates HR in *C. sinensis* similarly to generation in *C. limon* and *X. citri* 306 Δ*pthA4*, and Δ*pthA1,4*‐expressing PthA4^AT^ also behaves similarly in *C. sinensis* and *C. limon*, indicating that the same mechanisms of defence are triggered in both species (Fig. S8). Since the *C. sinensis* genome sequence is available (Wu *et al*., 2014; Xu *et al*., 2013) we used *C. sinensis* for molecular analysis of predicted targets (Grau *et al*., 2013). TALgetter consensus‐predicted sequences for PthA4^AT^ using *C. sinensis* promotorome are shown in Fig. 4a.

Although both consensus sequences described above are very similar, it is interesting to note that the second repeat of PthA4^AT^ (N*) binds preferably to T in the protein binding microarray (PBM) assay, whereas this position is predicted C/T by TALgetter. Moreover, the third repeat (NG), predicted T by TALgetter, binds equally to C and T in the PBM assay. In order to quantify the subtle differences in both consensus sequences in our PBM assay, we extracted signal intensities for the probes containing the sequence determined in PBM (TATYRCCTT) and the predicted by TALgetter (TAYTMCCTT). This analysis revealed a significantly higher binding of PthA4^AT^ to the sequence determined in PBM (TATYRCCTT). Nevertheless, both groups of sequences performed much better binding than the average array probes (Fig. 4b). The second repeat (N*) is shared between PthA4 and PthA4^AT^. Interestingly, the two known targets of PthA4 (*LOB1* and *SWEET1*; Hu *et al*., 2014) contain a T in this position, reinforcing the use of this high‐throughput methodology for experimental determination of DNA motifs recognized by TALEs.

**Figure 5:**
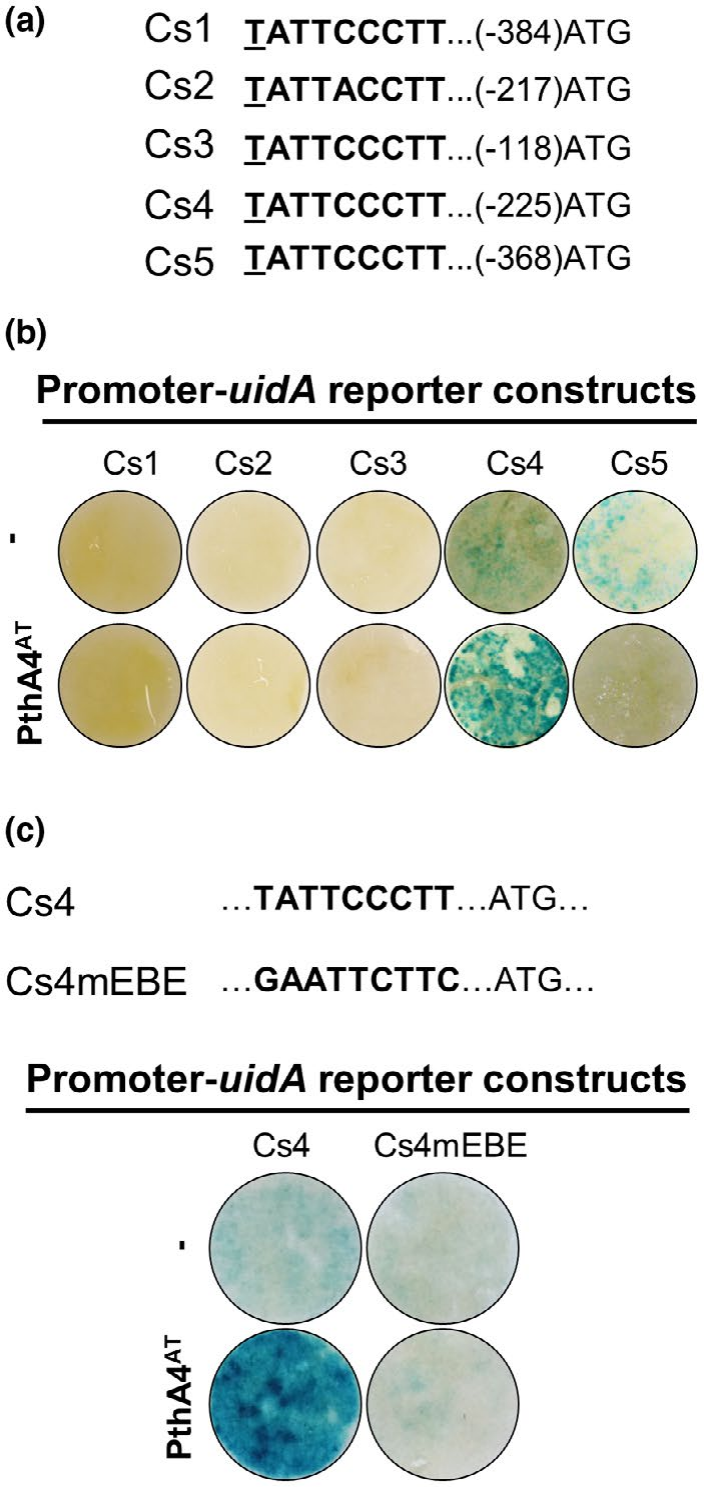
PthA4^AT^ activates transcription *in planta*. (a) Effector‐binding element (EBE) site of five *Citrus sinensis* (Cs) selected promoters. Underlined nucleotide indicates the 0 position of the EBE and the number in brackets indicates the position of last T nucleotide from the putative ATG. (b) GUS assay results after *Agrobacterium* co‐infiltration of Cs‐*uidA* promoters and 35S::PthA4^AT^. (c) EBE site and base mutation (mEBE) on Cs4. GUS staining in *Nicotiana benthamiana* leaves was performed 48 h post‐inoculation. Cs1, Cs2, Cs3, Cs4 and Cs5 correspond to *C. sinensis* IDs orange1.1g019568, orange1.1g047725, orange1.1g038742, orange1.1g048430 and orange1.1g048684, respectively.

To estimate the ability of PthA4^AT^ to activate transcription *in planta*, five putative target promoters of *C. sinensis* genes containing an EBE in their proximal region (400 bp upstream + 200 bp downstream of the transcription start site) were fused to the *uidA* (β‐glucuronidase [GUS]) reporter gene and transiently co‐expressed with the 35S promoter‐driven *pthA4^AT^* via *Agrobacterium* into *N. benthamiana* leaves. According to the different behaviour of *X. citri* A^T^ in *C. sinensis* and *C. clementina* (Chiesa *et al*., 2013), the five genes were selected to fulfil the criteria of being predicted targets of PthA4^AT^ by TALgetter (*P* value < 10^−5^) in *C. sinensis* but not in *C. clementine* and the consensus sequences obtained for PMB assay (Fig. 5a). As shown in Fig. 5b, transient expression assays indicate that PthA4^AT^ was able to activate GUS expression when this reporter was under the control of the Cs4‐promoter, indicating that the 7.5‐repeat TALE is able to bind DNA and activate transcription *in planta*. The other four promoters were not able to activate GUS PthA4^AT^‐dependent transcription, indicating that not all predicted binding sites are functional *in planta*. Next, to clarify if the PthA4^AT^‐dependent transcription of GUS under the Cs4 promoter was dependent on the EBE, we mutated this box in the *C. sinensis* promoter. As shown in Fig. 5c, the construct with the mutated EBE lost the ability to activate transcription of the *uidA* gene when co‐delivered with the 35S promoter‐driven *pthA4^AT^*, indicating that the PthA4^AT^‐mediating transcription observed for the Cs4 promoter is EBE‐dependent.

### HR development is mediated by PthA4^AT^‐dependent transcriptional activation

**Figure 6:**
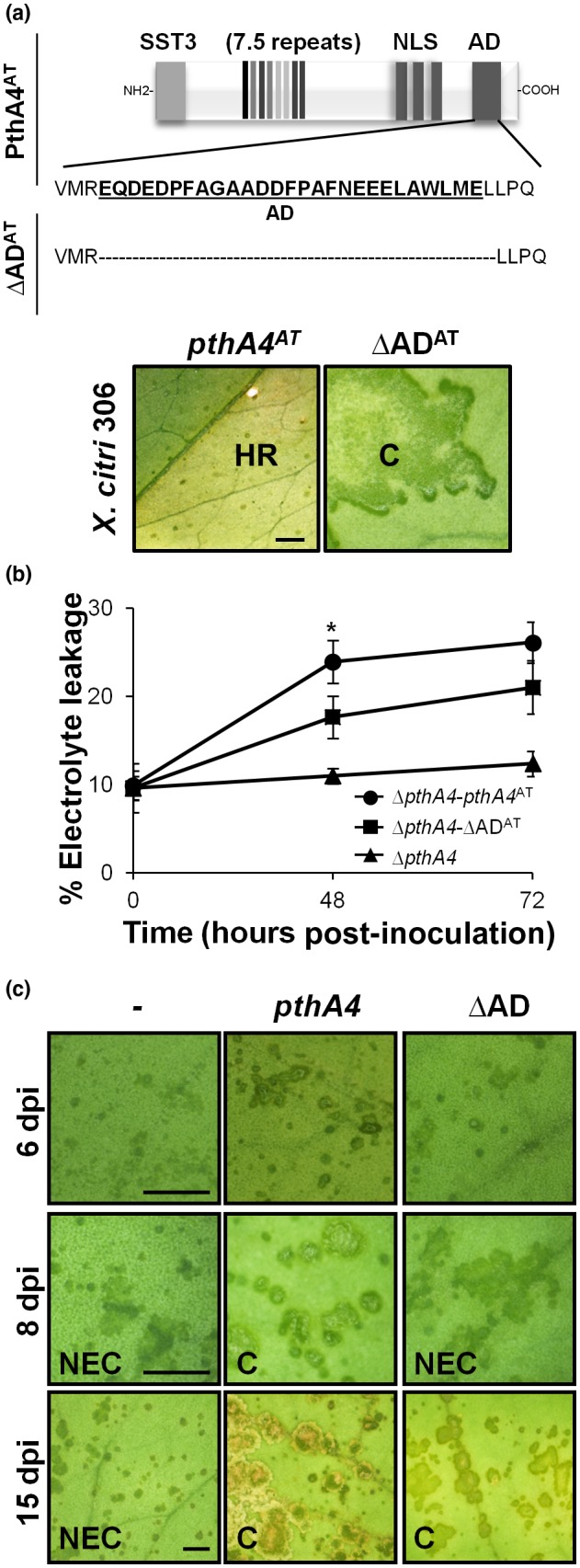
PthA4^AT^‐mediated resistance depends on transcriptional activation. (a) PthA4^AT^ variant harbouring a deletion of 27 amino acids on the activation domain (∆AD^AT^). Symptoms induced on *Citrus limon* leaves inoculated with *X. citri* 306 transformed with PthA4^AT^ or ∆AD^AT^. HR: hypersensitive response: C, canker; NLS, nuclear localization signal; AD, activation domain. (b) Percentage of electrolytic leakage at 0, 48 and 72 h post‐inoculation of *C. limon* inoculated with ∆*pthA4*, transformed with PthA4^AT^ or ∆AD^AT^. Values are expressed as means ±SD of three independent biological replicates. The dataset marked with an asterisk is significantly different as assessed by Tukey’s test (*P* < 0.05). (c) Symptoms induced on *C. limon* leaves inoculated with *∆pthA4* transformed with PthA4 or ∆AD from *Xanthomonas citri* 306 observed at different days post‐inoculation (dpi). ∆AD:PthA4 from *X. citri* 306 harbouring a deletion of 27 amino acids on the activation domain. NEC, non‐eruptive canker; C, canker. Scale bar: 10 mm.

The PthA4^AT^ protein presents an activation domain (AD) in its C‐terminal region, identical to the one in AvrBs3 protein from *Xanthomonas campestris* pv. *vesicatoria* (Szurek *et al*., 2001). To ascertain the relevance of transcriptional activation on the PthA4^AT^‐mediated defence response, a PthA4^AT^ deletion construct without the 27 amino acids marked in Szurek *et al*. (2001) as AD was generated (ΔAD^AT^). The expression of ΔAD^AT^ was unable to prevent canker development by *X. citri* wild‐type strains (Figs 6a and S9), suggesting that this activation domain is necessary for triggering an HR.

Interestingly, the expression of ΔAD^AT^ in Δ*pthA4*‐*X. citri* 306 strain showed a delay in the manifestation of HR after *C. limon* leaves inoculation, as assessed by conductivity assays. As shown in Fig. 6b, a significant reduction in electrolyte leakage was observed at 48 h after inoculation with ΔAD^AT^‐expressing Δ*pthA4* as compared with inoculation with Δ*pthA4* strain expressing the full‐length *pthA4^AT^* gene. As for the control, no cell death was observed after Δ*pthA4* inoculation. To establish whether the action of PthA4 as a virulence factor is similarly dependent on the activation domain, a deletion derivative of the *X. citri* 306 *pthA4* lacking the 27 amino acids of the activation domain (ΔAD) was generated and transformed into Δ*pthA4* mutant of *X. citri* 306. Interestingly, canker development was delayed after infection with Δ*pthA4‐*expressing ΔAD as compared with that containing the full‐length *pthA4* gene, as shown in Fig. 6c. Taken together, these results suggest that the deletion of AD in both *pthA4* and *pthA4^AT^* genes reduces, but does not completely abolish, gene activation, suggesting that HR development is mediated by PthA4^AT^‐dependent transcriptional activation, as a canonical TAL effector, presumably of an unknown executor *R* gene.

### PthA4^AT^ triggers hypersensitive response on non‐host *Nicotiana benthamiana*

**Figure 7:**
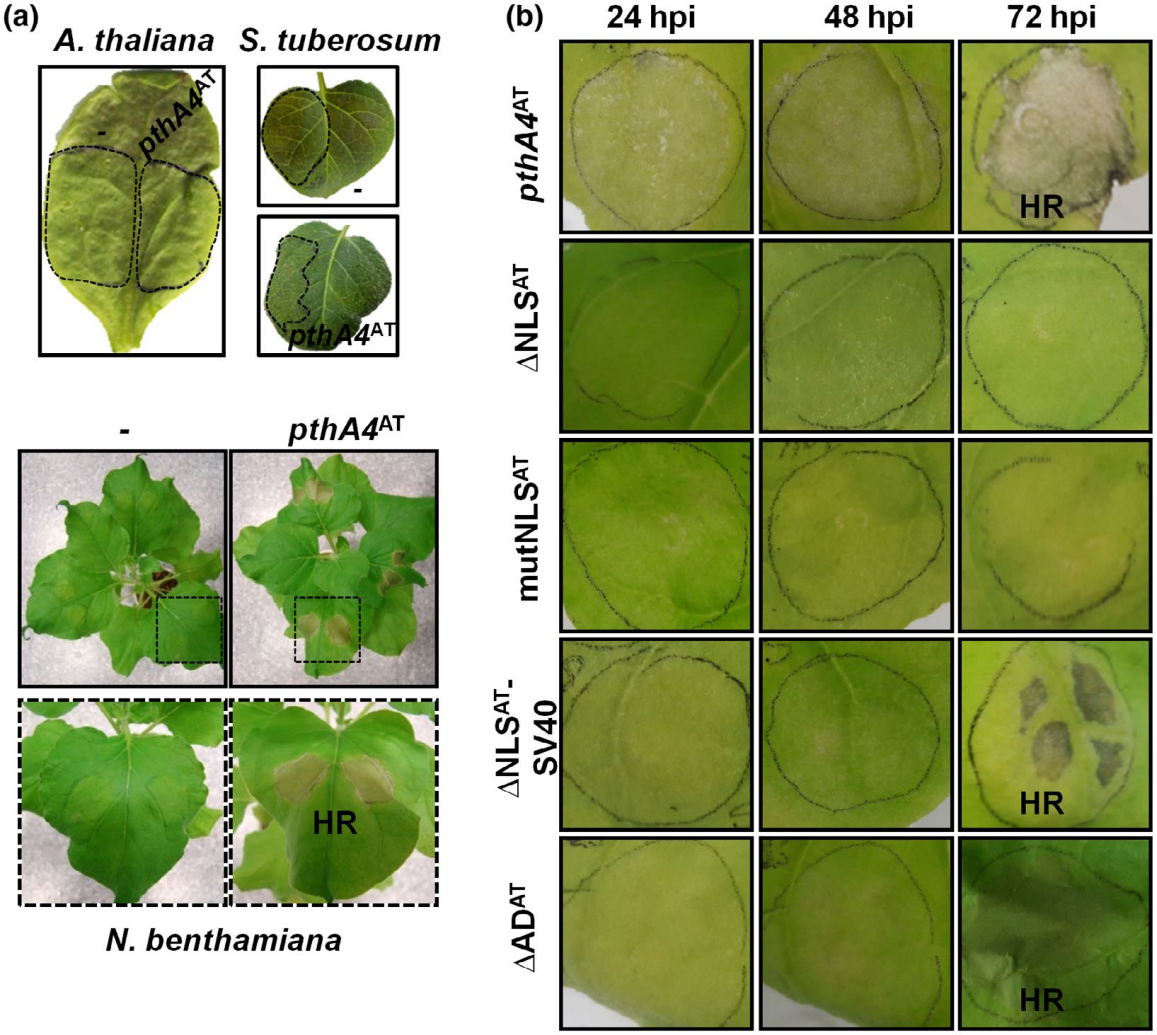
Hypersensitive response (HR) induced by PthA4^AT^ in non‐host *Nicotiana benthamiana* model plant. (a) *Agrobacterium tumefaciens* GV3101‐mediated transient expressions of PthA4^AT^ on *Arabidopsis thaliana*, *Solanum tuberosum* and *N. benthamiana* leaves. Leaves were agroinfiltrated and symptoms were photographed 72 h post‐inoculation (hpi). (b) Phenotypic response of the transient expression of the different PthA4^AT^ constructs evaluated in *N. benthamiana* leaves. Photographs were taken at 24, 48 and 72 hpi. ∆NLS^AT^:PthA4^AT^ derivative with an 83‐amino acid deletion in the C‐terminal region, eliminating the three conserved nuclear localization signals (NLS); mutNLS^AT^:PthA4^AT^ derivative mutated in the three NLS; ∆NLS^AT^‐SV40:∆NLS^AT^ derivative containing the NLS of SV40. ∆AD^AT^:PthA4^AT^ derivative with a 27 amino acid deletion in the activation domain (AD) region.

To study the effect of the expression of PthA4^AT^ on the activation of HR in non‐host plants, the TAL effector was analysed via *Agrobacterium*‐mediated transient expression in leaves of *Arabidopsis thaliana*, *Solanum tuberosum* and *N. benthamiana*, and plant reactions were scored over a 5‐day period. As shown in Fig. 7a, PhA4^AT^ was able to trigger a macroscopic cell death only on *N. benthamiana* leaves, suggesting that PthA4^AT^ induces an HR in this non‐host plant. To determine if this response required the C‐terminal domains of the protein, transient expression of PthA4^AT^ derivatives (∆NLS^AT^, mutNLS^AT^, ∆NLS^AT^‐SV40, ∆AD^AT^) was assessed. Similar to citrus, ∆NLS^AT^ and mutNLS^AT^ constructs did not develop a visible HR and ∆NLS^AT^‐SV40 partially restored the cell death response (Fig. 7b). This suggests that, as with citrus, PthA4^AT^ action in *N. benthamiana* requires localization to the nuclei. Moreover, the full HR manifestation required the presence of a functional AD (Fig. 7b), suggesting that in this non‐host plant PthA4^AT^ is acting as a canonical TAL effector.

## Discussion

Surveys of the size of TALEs in nature indicate that the great majority of them contain a mean of 18.5 RVDs, indicating that this length has been selected by evolution to achieve high target specificity (Boch and Bonas, 2010; Rinaldi *et al*., 2017). The presence of very short TALEs in some bacterial strains has been documented, as exemplified by the 1.5‐repeat AvrXa3 of *Xanthomonas oryzae* pv. *oryzae* (*Xoo*) (Wu *et al*., 2006), but they are likely non‐functional for transcriptional activation (Boch *et al*., 2009). These short TALEs are probably by‐products of the complex sequence rearrangement that shapes the TALomes of the different strains (Denancé *et al*., 2018). For TALEs containing 6.5 to 9.5 RVDs, transcriptional activation is still possible, although it is much weaker than for longer RVDs (Boch *et al*., 2009). This raises the question of whether these intermediate‐length TALEs are biologically functional or not, given that their shorter length will translate into an increased number of potential targets, which in addition will presumably not be highly expressed (Richter *et al*., 2016). Tn*5*‐based mutagenesis in *Xoo* generated a mutant with an insertion in a gene encoding a TALE with 8.5 RVDs; the mutation conferred increased avirulence to rice cultivars carrying the *Xa3 R* gene (Li *et al*., 2004). However, the effect was maintained in a second mutant expressing a 124 amino acid open reading frame containing only the NLS and the C‐terminal activation domains. These results suggested that the effect was not dependent on the RVD, but rather on the recognition of the C‐terminal part of the protein by Xa3, in a similar way to Bs4 and Xo1 R proteins in pepper and rice, respectively (Schornack *et al*., 2004; Triplett *et al*., 2016). In the current study, the evidence suggests that the avirulence of PthA4^AT^ on *C. limon* and *C. sinensis* is dependent upon the protein entering the nucleus activating transcription of an executor *R* gene. As was demonstrated, nuclear localization and transcriptional activation are necessary for triggering a proper HR (Figs 3 and 6). To our knowledge, this is the first case where a ‘short’ 7.5‐repeat TALE exerts a biological function via the RVD and transcriptional activation, i.e. in the manner of a classical TALE.

The C‐terminal region of all members of the PthA/AvrBs3 family contains an acidic amino acid domain, a property typical of eukaryotic acidic transcription AD. This motif is required for avirulence activity of the AvrXa10 protein in rice (Zhu *et al*., 1998, 1999) and of the AvrBs3 protein in pepper (Szurek *et al*., 2001). When the same 27 amino acids critical for avirulence of AvrBs3 were removed from PthA4^AT^ (to give the ΔAD^AT^ derivative), its ability to restrict canker development was lost, indicating the importance of this activation domain for PthA4^AT^ avirulence (Fig. 6b). However, when ΔAD^AT^ is expressed into the Δ*pthA4* mutant strain, an HR is still developed, although it is retarded, suggesting that the deletion in the activation domain did not completely abolish gene activation. Interestingly, similar results were obtained in the non‐host *N. benthamiana* (Fig. 7b). Although activation domains are characterized by the presence of acidic amino acids, acidity is not the sole characteristic of an activating region and the net negative charge does not strictly correlate with the efficiency (Gill *et al*., 1990). Powerful artificially generated transcriptional activation domain completely devoid of acidic residues has been generated (Lu *et al*., 2000). It is thought that the role of these domains is merely to stick to the transcriptional machinery and thereby to recruit it to DNA (Ptashne and Gann, 1997; Yuan *et al*., 2016). There are data from other TALEs with nearly identical C‐terminal regions that support our results. In AvrBs3, the deletion of the 27 amino acids AD does not avoid transcriptional activation in yeast (Szurek *et al*., 2001) and similar results were observed for the AvrXa10 protein of *Xoo* (Zhu *et al*., 1998). Moreover, only 50% reduction in transcriptional activity was observed when the 28 amino acids immediately upstream of the NLS and AD domain of *X. campestris* pv. *armoraciae* Hax3 protein were truncated (Zhang *et al*., 2011). In these three truncated‐AD TALEs, and in ∆AD *pthA*s as well (Fig. 6), this stretch still contains 21% of acidic amino acids that could make the protein stick to the transcriptional machinery.

The C‐terminal portion of PthA also encodes three NLS that are critical for localization to the host cell nucleus (Yang and Gabriel, 1995). It was demonstrated that PthA4 TALE targeted to the nucleus by interaction with different host nuclear factors (Domingues *et al*., 2010; Soprano *et al*., 2013; de Souza *et al*., 2012). Using a dominant‐negative strategy, Yang *et al*. (2011) generated transgenic *C. sinensis* resistant to canker by overexpressing the NLS of PthA4, this NLS playing an importin‐binding interference role. It could be argued that the mechanism by which PthA4^AT^ exerts its role in pathogenic *X. citri* strains is similar, and that ∆NLS^AT^ and mutNLS^AT^ derivatives lost their ability to generate the HR only because they cannot interfere anymore with the host importins. However, this is unlikely, as expression of PthA1^AT^ (with identical NLS region) on *X. citri* strains does not influence canker development after infection of *C. limon*, and PthA1^AT^ expression on the Δ*pthA1,4* or Δ*pthA1* mutants did not cause HR (Fig. 2). All this evidence suggests that canker protection caused by PthA4^AT^ goes via a different mechanism, presumably via the activation of an *R* executor gene. Until now, only five of those genes have been identified (Zhang *et al*., 2015). They trigger host responses associated with HR, similarly to classical *R* genes like receptor like‐kinases (RLK) and nucleotide binding site‐leucine‐rich repeat (NBS‐LRR) genes, but if the resistance pathways triggered by the TALE‐mediated executor *R* genes intersect with those of the other *R* genes remain unknown. *Agrobacterium*‐mediated delivery of a 35S promoter‐driven executor Bs3 coding‐region did not cause a visible HR in citrus (Shantharaj *et al*., 2017). The functionality of the other known executor R proteins in citrus is unknown. We could not find homologous to the known executor *R* genes on the TALgetter predicted PthA4^AT^ target genes list (Table S3). This would suggest a different mechanism for PthA4^AT^‐induced HR activation in citrus.

*X. citri* A^T^ can still develop canker lesions without the presence of the *pthA4* virulence gene in *C. clementina* and *X. aurantifolia* (Chiesa *et al*., 2013). This indicates that the absence of canker in *X. citri* A^T^‐infected *C. limon* is not solely an effect of the absence of the *pthA4* gene. In support of this contention, reintroduction of the *pthA4* virulence gene into *X. citri* A^T^ does not restore canker development in *C. limon.* Finally, the host specificity observed for *X. citri* A^T^ weakens the hypothesis that PthA4^AT^ could be acting indiscriminately over many gene targets, generating a cytotoxic effect by the serendipitous activation of some or many of them, leading to metabolic perturbation as suggested by Reyon *et al*. (2012). This is unlikely, as predictions by TALgetter indicate that the number of potential targets for such a short TALE is huge not only in *C. sinensis*, where the HR is observed, but also in the susceptible *C. clementine*; many of the targets are coincident (Tables S3 and S4). As expected, we could determine that not all the potential targets will be activated by PthA4^AT^ (Hummel *et al*., 2012; Pereira *et al*., 2014). In fact, we could obtain transcriptional activation in only one out of the five promoters tested (Fig. 5b). This confirms that PthA4^AT^‐dependent transcriptional activation is possible, but suggests a gene (or genes)‐specific activation preference that requires a more thorough approach to decipher. The generation of artificial PthA4^AT^ derivatives, using combinatorial cloning (Geiβler *et al*., 2011) coupled with pathogenicity tests on *C. limon* and non‐host *N. benthamiana*, could help to define PthA4^AT^ specificity and thereafter to narrow down the number of targets in the search for those triggering the defence response. Transcriptome profiling using *X. citri* strains containing the different derivatives of PthA4^AT^ generated in this study will support the criterion for the identification of the *R* genes (Boch *et al*., 2014; Strauss *et al*., 2012). Given the short length of PthA4^AT^, a specific pattern of activation across multiple targets, as proposed for the 18‐repeat *Tal2a* gene that elicits HR in rice (Hummel *et al*., 2017), is plausible.

Any selective advantage was found for *X. citri* A^T^ in any of the hosts assayed (Chiesa *et al*., 2013). The existence of the *pthA4^AT^* gene is perhaps just an accident, a by‐product of a recombination event in the course of effector evolution. Interestingly, Ye and colleagues (2013) showed that two further strains of *X. citri* (029‐2 and 049) with attenuated virulence and reduced bacterial growth *in planta* were characterized by the absence of the 3.4‐kb band characteristic of the *pthA4* gene, and by the appearance of a smaller band of around 2.4 kb, in a similar way to *X. citri* A^T^ (Fig. 1). It would be interesting to isolate and sequence those genes, and test if they also can confer avirulence on citrus hosts. Recently, Pacbio sequencing of *X. citri* strains also revealed a 6.5‐repeat *pthA1* variant in the LM180 strain, obtained from infected grapefruit samples (Gochez *et al*., 2018), but the biological relevance of this TALE gene is unknown. The discovery of a TALE conferring canker resistant in *C. limon* and *C. sinensis* paves the way to identify citrus *R* genes involved in this important aspect of citriculture.

## Experimental Procedures

### Bacterial strains growth conditions, plant material and inoculation assays

The strains and plasmids used in this study are listed in Tables S1 and S2, respectively. *X*. *citri* strains were cultured at 28 °C with shaking in peptone yeast and malt extract (PYM) medium (Cadmus *et al*., 1976). *Escherichia coli* strains DH5α and BL21 and *Agrobacterium tumefaciens* GV3101 were grown in Luria‐Bertani (LB) medium at 37 °C and 28 °C, respectively. Recombinants plasmids were introduced into the *X. citri* and *Agrobacterium* strains by electroporation (Roeschlin *et al*., 2017). When required, the antibiotics ampicillin (100 µg/mL), kanamycin (50 µg/mL), rifampicin (50 µg/mL) or spectinomycin (100 µg/mL) were added to the growth media.

‘Eureka’ lemon [*C. limon* (L.) Burm. f.] grafted onto Troyer citrange, ‘Valencia Late’ sweet orange [*C. sinensis* (L.) Osbeck] grafted onto citrange Carrizo, *Arabidopsis thaliana* ecotype Col‐0, *Solanum tuberosum* ‘Spunta’ and *N. benthamiana* plants were grown under controlled conditions in a growth chamber with a temperature of 25–27 °C and photoperiod of 16 h light/8 h dark. For pathogenicity assays on *Citrus* spp., new shoots were selected according to Roeschlin *et al*. (2017). Bacterial suspensions of 10^7^ or 10^9^ colony‐forming units (cfu) per millilitre were prepared in 10 mM MgCl_2_ and inoculated by infiltration or cotton swab onto 20‐day‐old leaves of the new shoots, respectively. The plants were maintained for 15 days in a growth chamber. Symptoms progression was phenotypically monitored using an MVX10 stereomicroscope as reported in Roeschlin *et al*. (2017) and conductivity was measured according to Chiesa *et al*. (2019). For *Agrobacterium*‐mediated transient expression, strains preparation and induction were conducted as described by Enrique *et al*. (2011).

### Southern hybridization analysis

Plasmid DNA from *X. citri* strains was isolated according to the manufacturer's instructions (QIAGEN Plasmid Midi Kit; QIAGEN, Mainz, Germany). DNA (10 μg) was digested with *Eco*RI or *Bam*HI overnight at 37 °C and subjected to electrophoresis on 0.8% agarose gel. DNAs were transferred to Hybond N+ nylon membrane (Amersham International, UK) using standard protocols (Sambrook *et al*., 1989). The blots were hybridized with a non‐radioactive labelled DNA probe (*pthA*), generated by a polymerase chain reaction (PCR) product, using the primer pairs Jpth1/Jpth2 (Cubero and Graham, 2002). Probe labelling and signal detection were performed according to the manufacturer's instructions (AlkPhos DIRECT kit, GE Healthcare UK Limited Amersham, Little Chalfont, Buckinghamshire, UK).

### Cloning and sequencing of TALE genes

The repeat region of *pthA* genes was cloned as a *Bam*HI fragment into pUC18 vector (ThermoFisher Scientific, Waltham, MA, USA), screened by PCR with Jpth1/Jpth2 primers and Sanger sequenced (DNA Sequencing Facility, University of Maine, USA). To obtain the 5ʹ and 3ʹ terminal regions of PthAs, plasmids from *X. citri* A^T^ were sequenced using Roche (454) pyrosequencing technology. Reads were assembled with Newbler v. 2.6 software (Roche 454 Life Sciences, Branford, CT, USA). The final sequence of the two circular plasmids was identical to the reference plasmids (pXAC64 and pXAC33) from *X. citri* strain 306 (da Silva *et al*., 2002). PthAs sequences were deposited at GenBank under the accession numbers MK425208, MK425209, MK425210 and MK425211.

To obtain full‐length *pthA* genes, *Bam*HI‐fragments were subcloned into pUC57‐RR linearized *Bam*HI vector (Data S1) and then screened by PCR and sequenced to check orientation (pUC57RR‐*pthA*). Constructs with NLS deletion (∆NLS), NLS mutations (mutNLS), SV40 insertion (SV40) and AD deletion (∆AD) were obtained by replacing the *Bcl*I/*Hin*dIII 3ʹ terminal sequence of pUC57RR‐*pthA* with the corresponding fragments obtained by gene synthesis (Data S1).

For *X. citri* and *A. tumefaciens* expression, *pthA* constructs from pUC57RR were subcloned into the pBBR1‐MCS2 or pCHF3 vectors using the restriction sites *Xho*I/*Hin*dIII or *Xba*I/*Hin*dIII, respectively.

### Immunoblot analysis

*Xanthomonas citri* overnight cultures (200 μL) were centrifuged and resuspended in protein sample buffer using a volume equal to the optical density at 600 nm 10^‐1^. Equal volumes (15 µL) of these whole‐cell extracts were separated by 10% SDS‐PAGE and transferred to polyvinylidene fluoride membranes (PVDF‐Immun‐Blot®, BioRad, Hercules, CA, USA). Immunoblotting was performed using 3×FLAG antibodies (1:5000 dilution, Cat# F3165, Sigma‐Aldrich, St Louis, MO, USA) and visualized using a peroxidase‐conjugated goat anti‐rabbit IgG (1:6000 dilution) and Pierce™ ECL Western Blotting Substrate (ThermoFisher Scientific, Waltham, MA, USA) according to manufacturer’s instructions.

### Protein binding microarrays

DNA corresponding to full‐length *pthA4^AT^* was transferred to pMAL‐c2 vector (New England Biolabs, Inc., MA, USA), yielding maltose binding protein (MBP) N‐terminal fusions. MBP–*pthA4^AT^* constructs were transformed into *E. coli* BL‐21 strains and selected with the corresponding antibiotic. Cultures with an optical density at 600 nm of 0.8 were incubated at 37 °C for 15 min with 2 mM betaine monohydrate (Sigma‐Aldrich) and then induced for 2 h with 0.5 mM isopropyl β‐d‐1‐thiogalactopyranoside (Sigma‐Aldrich). Expression of MBP‐*pthA4^AT^* was analysed by Coomassie blue staining of standard SDS‐PAGE and recombinant protein extracts were obtained from 25 mL of induced cultures. The PBM was performed as described previously (Godoy *et al*., 2011). Normalization of probe intensities and calculation of *E*‐scores and *Z*‐scores of all of the possible 8‐mers were carried out with the PBM Analysis Suite. To obtain a single representative motif, we followed the method ‘PWM_align_E’, in which the 9‐mer motifs with *E*‐score > 0.45 were aligned and each sequence in the alignment is first weighted by the *E*‐score of the corresponding sequence. Then, the positions present in at least half of the sequences in the alignment were considered, and the resulting alignment is converted to a position frequency matrix with Enologos (http://www.benoslab.pitt.edu/cgi-bin/enologos/enologos.cgi).

### TALgetter predictions in *Citrus* spp. and GUS assay

PthA4^AT^ binding elements were searched in *C. sinensis* and *C. clementina* promoterome (400 bp upstream + 200 bp downstream of the transcription start site) by using TALgetter software and considering both strands (http://galaxy2.informatik.uni-halle.de:8976/; Grau *et al*., 2013; Streubel *et al*., 2017). The candidate promoters are listed in Tables S3 and S4. Selected promoter regions were amplified from the corresponding genomic DNA using designed primers (Table S5). The promoter fragments were digested with the corresponding restriction enzyme (*Bam*HI or *Xba*I with *Nco*I) and fused with *uidA* (GUS) gene in pCAMBIA1303 expression vector. Positive clones were analysed by PCR amplification and DNA sequencing, and transformed into *A. tumefaciens* GV3101. EBE‐mutated Cs4 promoter was generated by overlapping PCR using primers incorporating mutations in the EBE site (Table S5).

For GUS expression in *N. benthamiana* leaves, two *Agrobacterium* suspensions were mixed in a ratio of 1:1. Forty‐eight hours post‐infiltration, leaf disks were stained on GUS staining buffer (50 mM NaPO_4_ (pH 7.0), 0.1% Triton X‐100, 10 mM EDTA, 1 mM K_3_Fe(CN)_6_, 1 mM K_4_Fe(CN)_6_, 0.5 mg/mL X‐gluc] and incubated at 37 °C for 3 h. Then, the disks were cleared in ethanol and photographed.

## Accession Numbers

MK425208, MK425209, MK425210 and MK425211.

## Supporting information


**Fig. S1** Alignment of PthA1 from *X. citri *A^T ^and *X. citri *306.

**Fig. S2** Alignment of PthA4 from *X. citri *A^T ^and *X. citri *306.

**Fig. S3** Western‐blot analysis of PthA expression on *X. citri *strains.

**Fig. S4** Macroscopic symptoms developed by *X. citri *A^T^ and *X. citri *306 ∆*pthA4* mutant expressing *pthA4*.

**Fig. S5** Phenotypic response of PthA1^AT^ and PthA4^AT^ expressed in Argentinian *X. citri *T strain in *Citrus limon *leaves.

**Fig. S6** Mutations in the PthA4^AT^ C‐terminal region.

**Fig. S7** Phenotypic response of *X. citri *306 strain expressing mutant version in nuclear localization of PthA4^AT^.

**Fig. S8** PthA4^AT^ triggers host defense response in *Citrus sinensis *leaves.

**Fig. S9** Phenotypic response on *C. limon *leaves inoculated with *X. citri *T transformed with ∆AD^AT^.

**Table S1.** Bacterial strains.

**Table S2.** Plasmids used in this study.

**Table S3.** TALgetter results of candidate promoters to PthA4^AT^ on *C. clementine*.

**Table S4.** TALgetter results of candidate promoters to PthA4^AT^ on *C. sinensis*.

**Table S5.** List of oligonucleotide primers used in this study.

**Table S6.** List of motifs variants obtained with PBM11 protein‐binding microarrays for PthA4^AT^.

**Data S1.** Design sequences for synthesis and cloning of the full‐length of *pthAs* and mutant construction.
