# Supplementary figures and images for: PthA4AT, a 7.5‐repeats transcription activator‐like (TAL) effector from Xanthomonas citri ssp. citri, triggers citrus canker resistance
Source: Mol Plant Pathol. 2019 Jul 5;20(10):1394–407. doi: 10.1111/mpp.12844 (PMC6792138; doi:10.1111/mpp.12844)

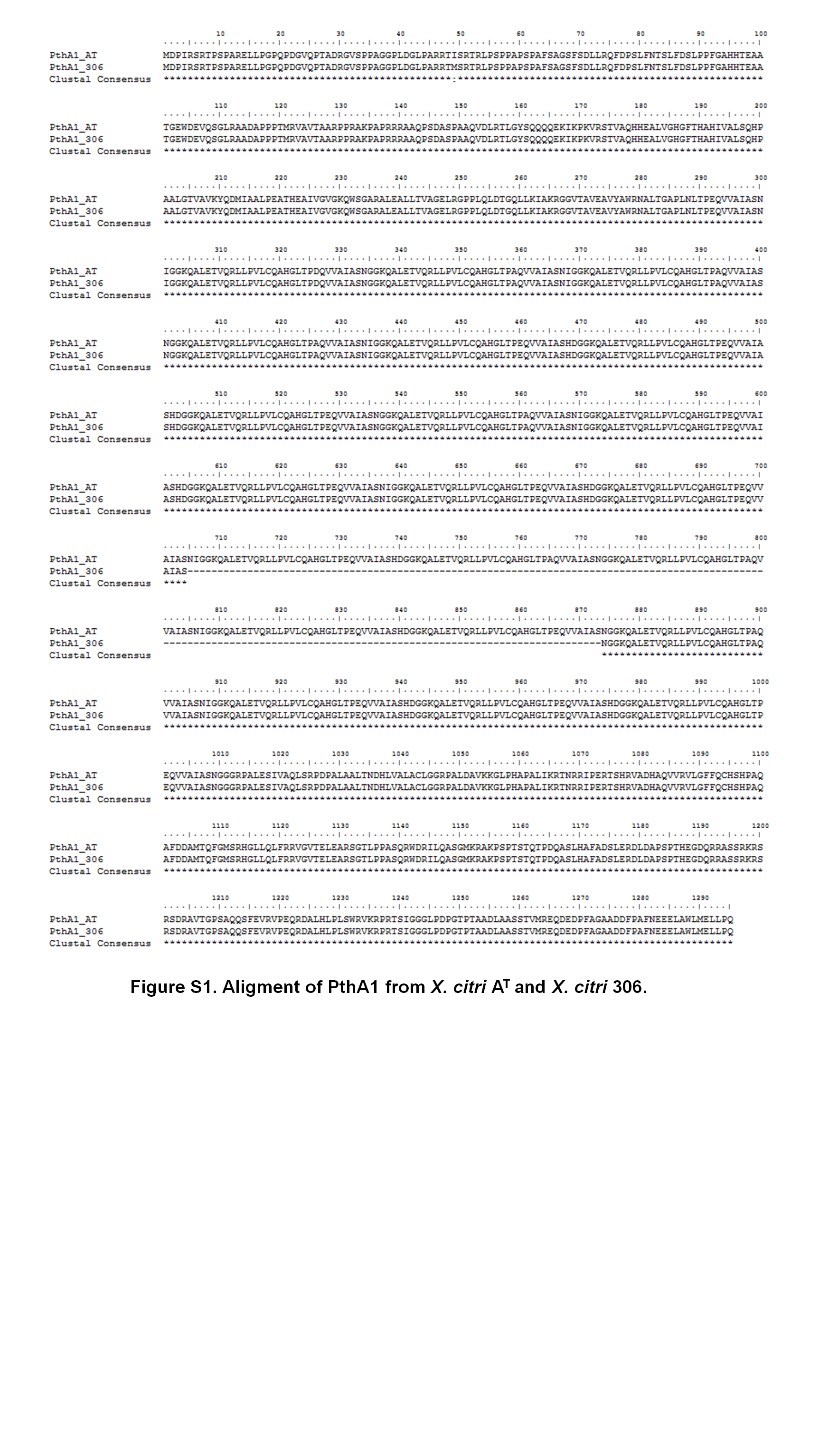

Supplement: Supplementary file 1 — Fig. S1 Alignment of PthA1 from X. citri AT and X. citri 306. [file MPP-20-1394-s001.tif]

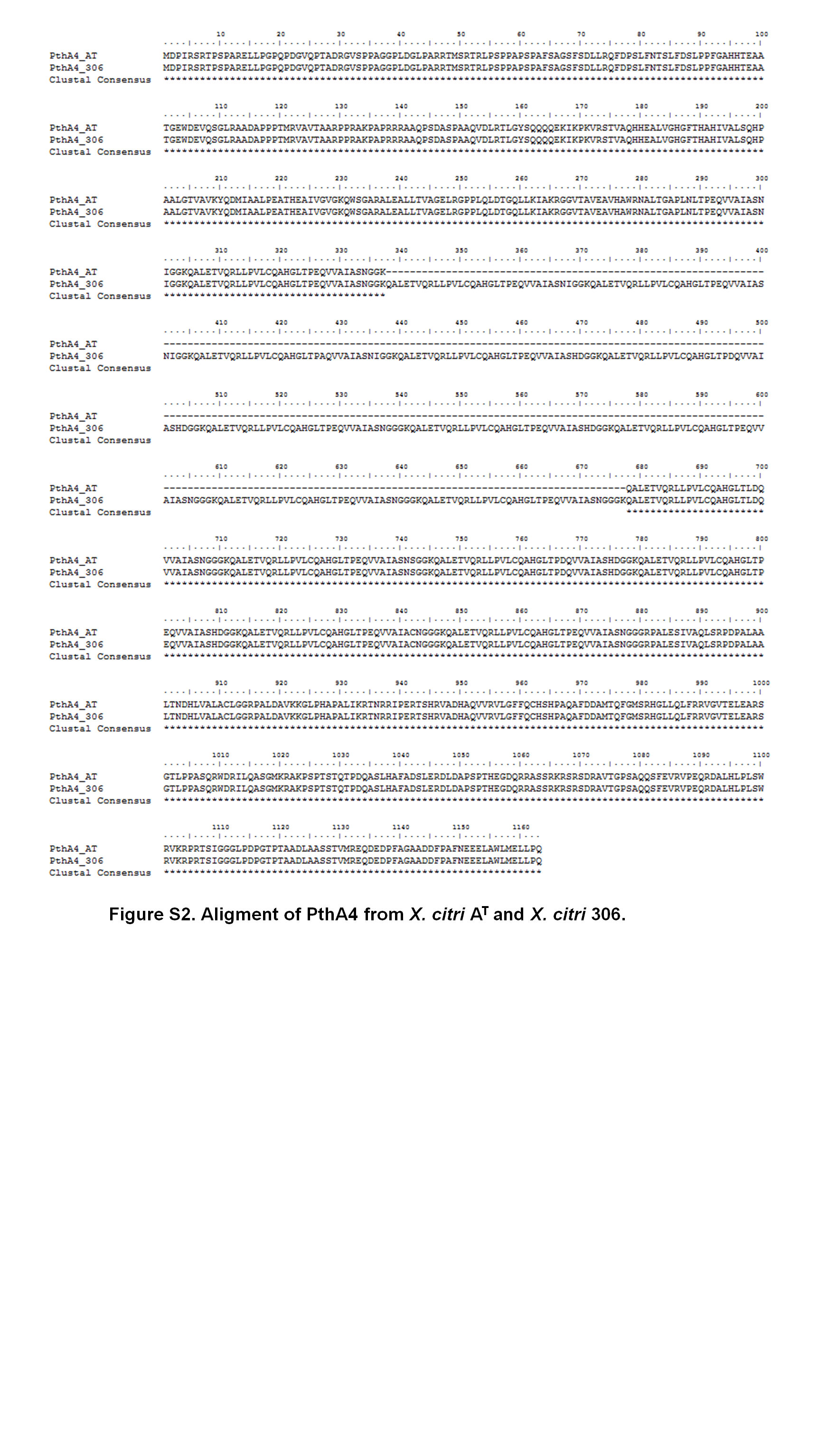

Supplement: Supplementary file 2 — Fig. S2 Alignment of PthA4 from X. citri AT and X. citri 306. [file MPP-20-1394-s002.tif]

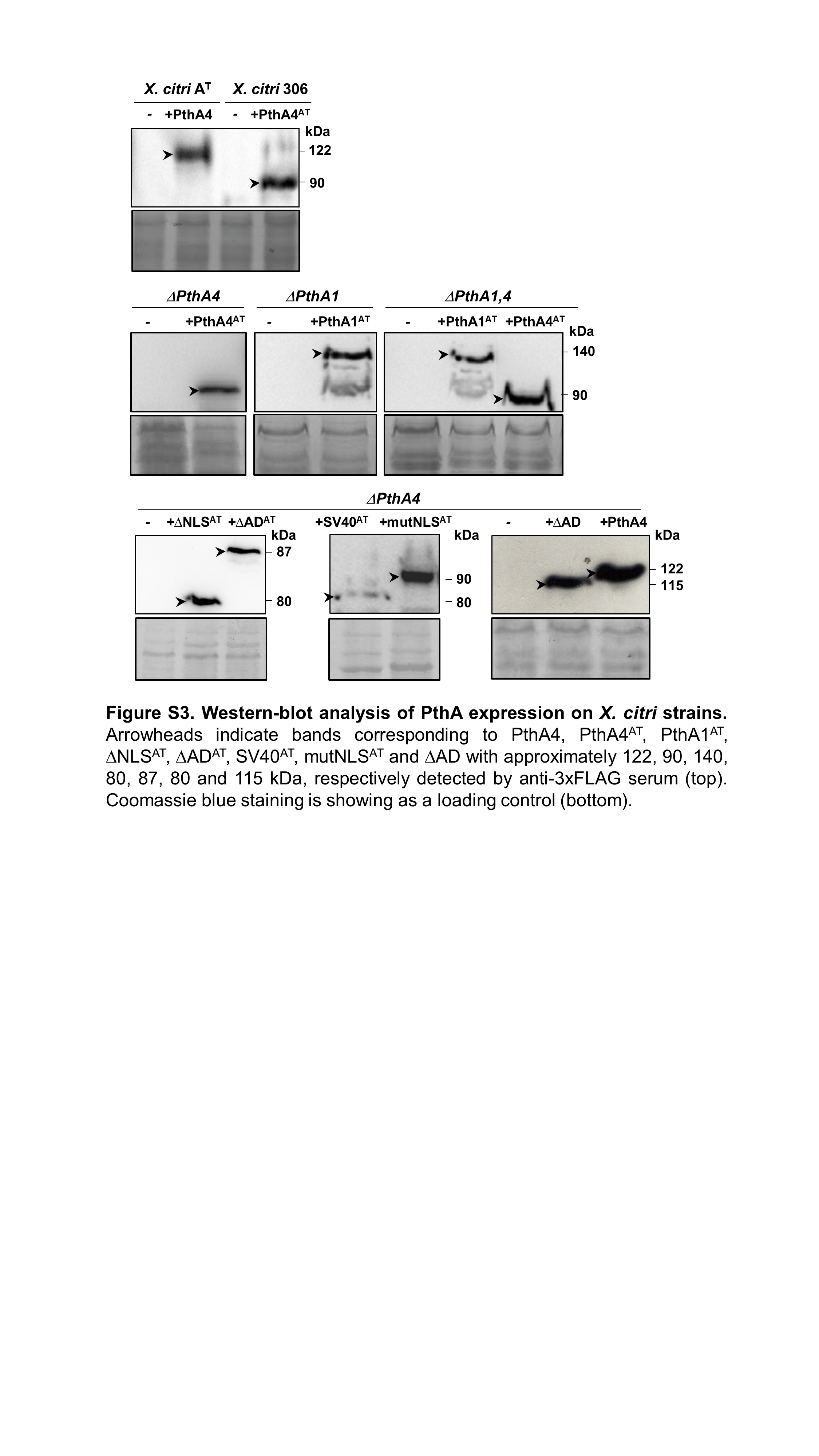

Supplement: Supplementary file 3 — Fig. S3 Western‐blot analysis of PthA expression on X. citri strains. [file MPP-20-1394-s003.tif]

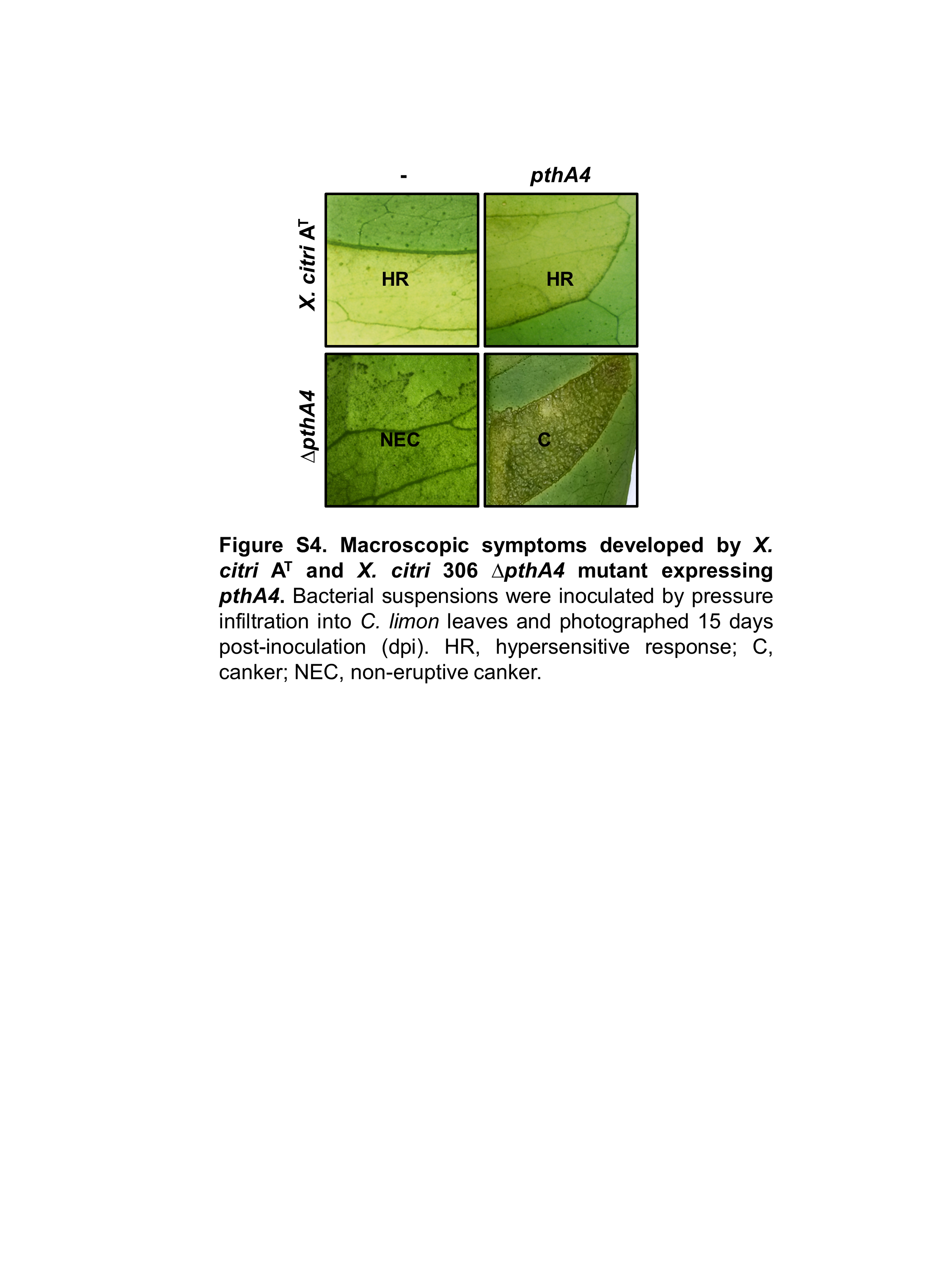

Supplement: Supplementary file 4 — Fig. S4 Macroscopic symptoms developed by X. citri AT and X. citri 306 ∆pthA4 mutant expressing pthA4. [file MPP-20-1394-s004.tif]

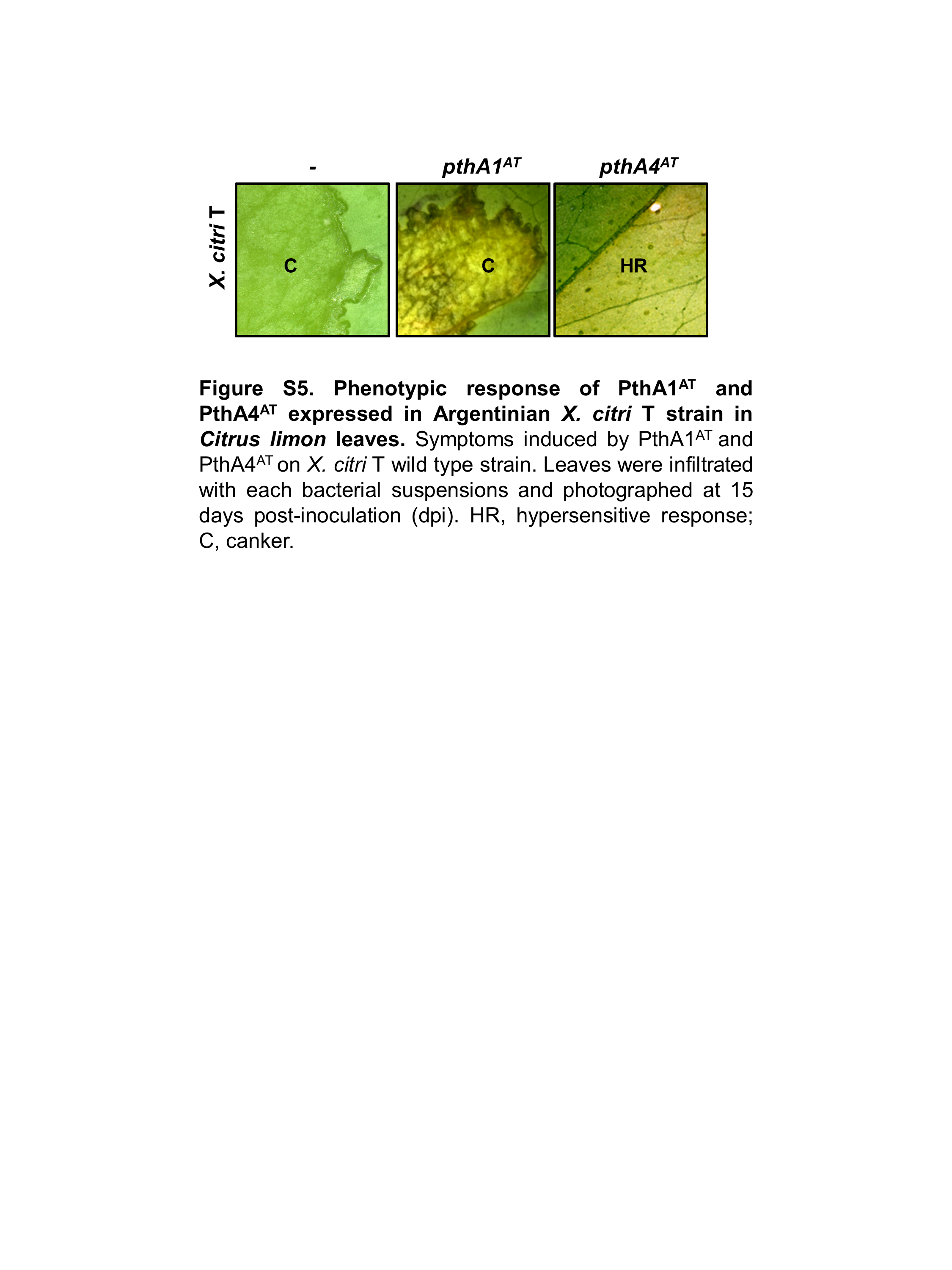

Supplement: Supplementary file 5 — Fig. S5 Phenotypic response of PthA1AT and PthA4AT expressed in Argentinian X. citri T strain in Citrus limon leaves. [file MPP-20-1394-s005.tif]

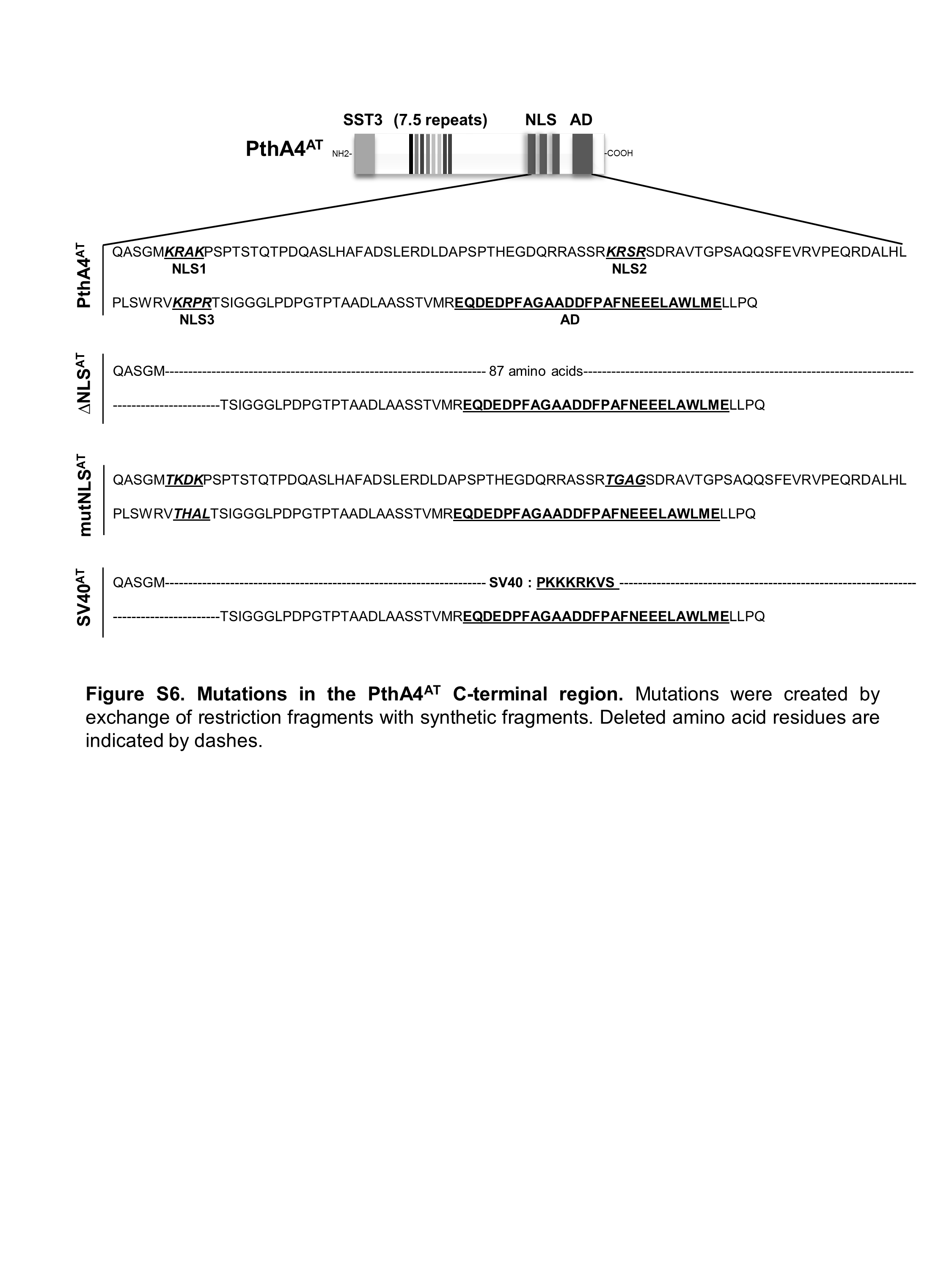

Supplement: Supplementary file 6 — Fig. S6 Mutations in the PthA4AT C‐terminal region. [file MPP-20-1394-s006.tif]

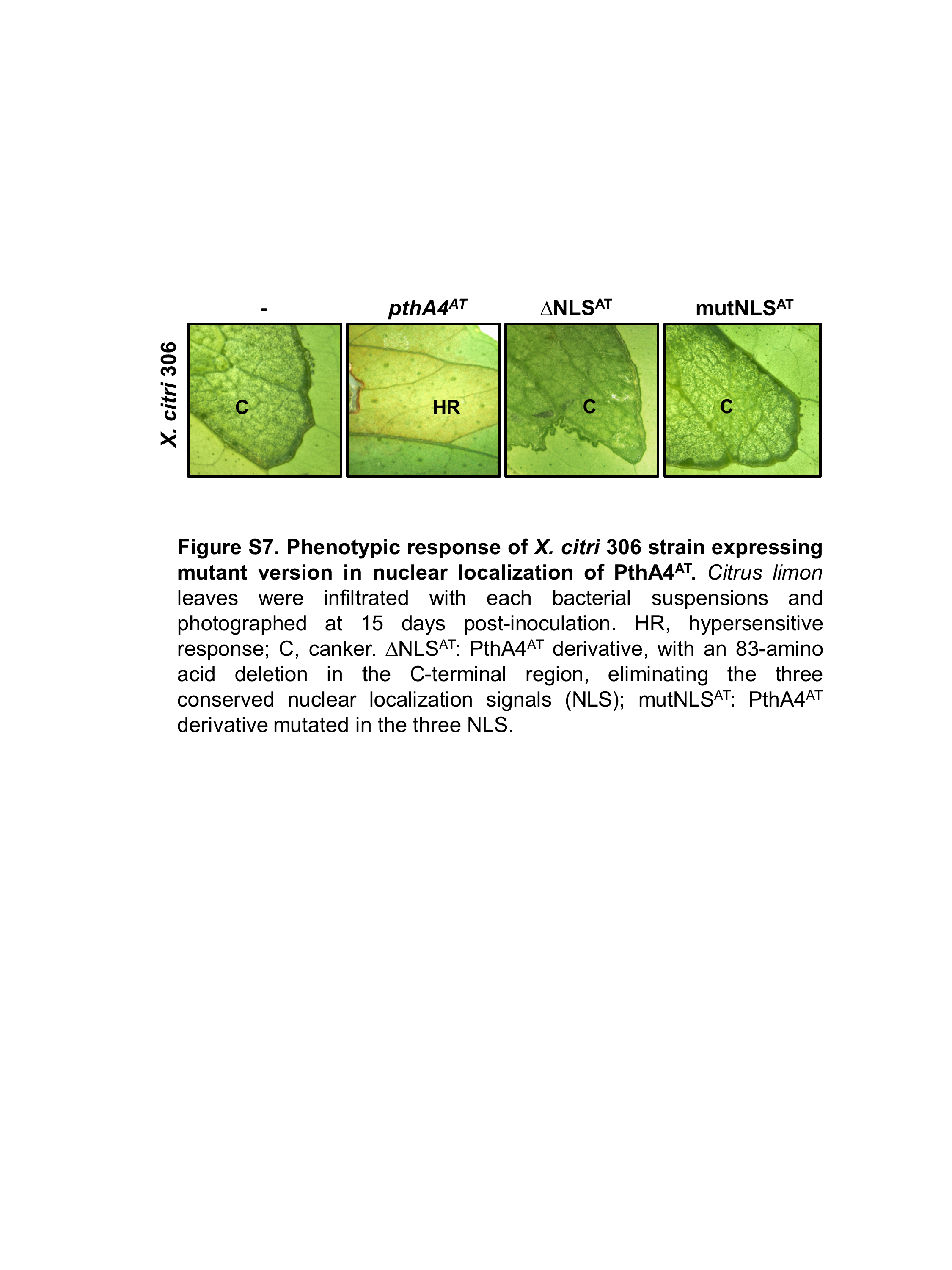

Supplement: Supplementary file 7 — Fig. S7 Phenotypic response of X. citri 306 strain expressing mutant version in nuclear localization of PthA4AT. [file MPP-20-1394-s007.tif]

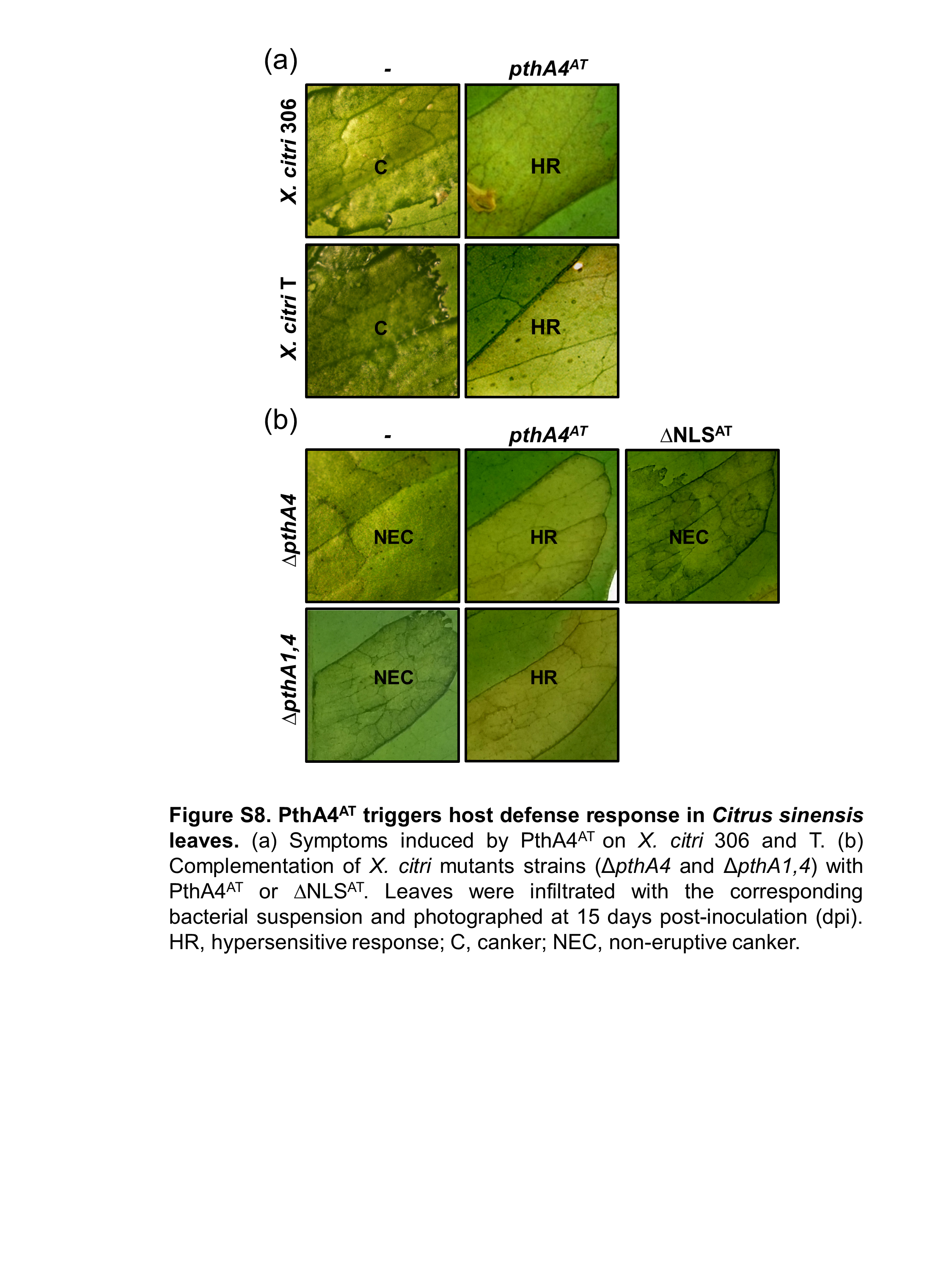

Supplement: Supplementary file 8 — Fig. S8 PthA4AT triggers host defense response in Citrus sinensis leaves. [file MPP-20-1394-s008.tif]

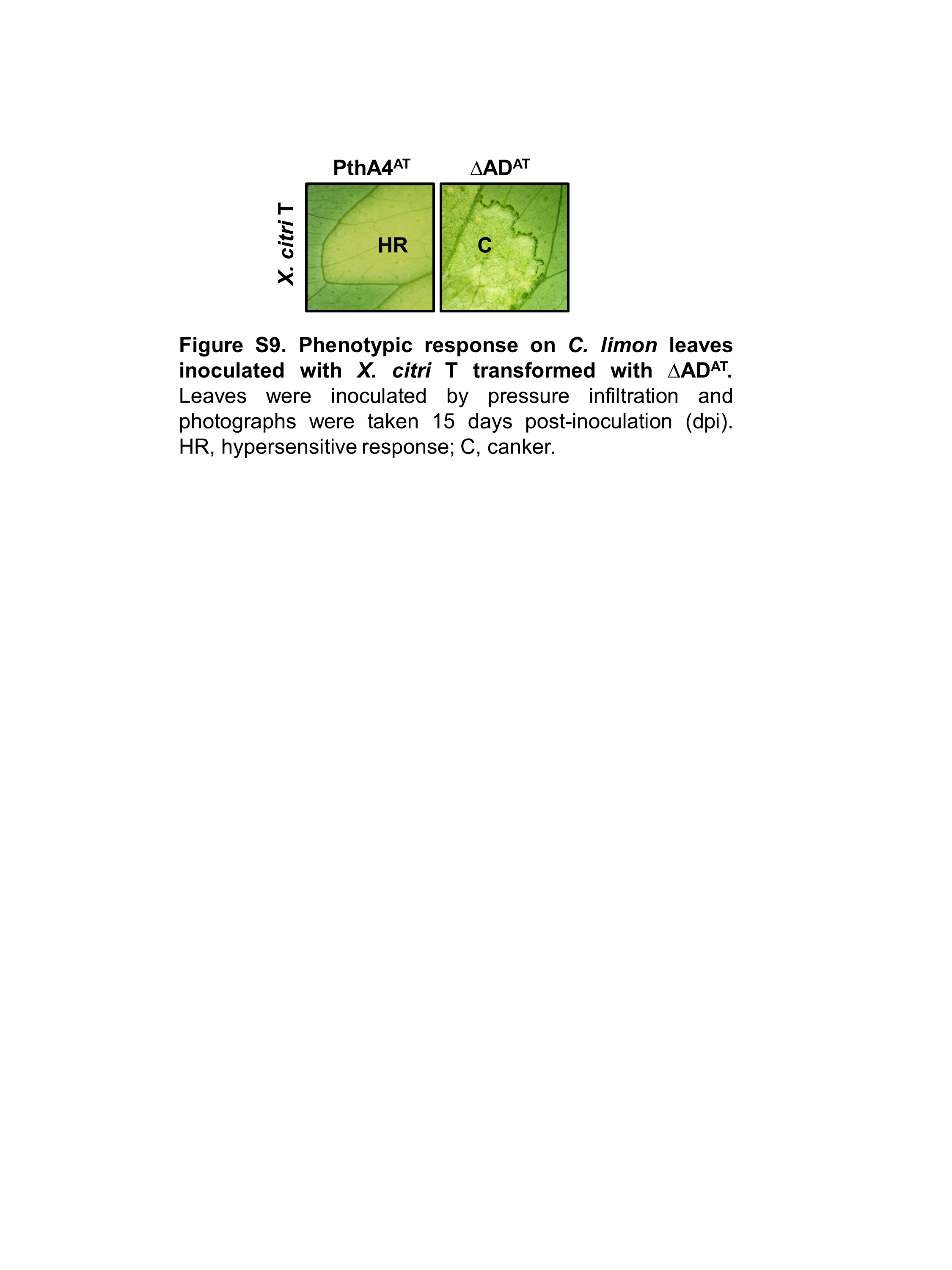

Supplement: Supplementary file 9 — Fig. S9 Phenotypic response on C. limon leaves inoculated with X. citri T transformed with ∆ADAT. [file MPP-20-1394-s009.tif]
