# Supplementary material for: PthA4AT, a 7.5‐repeats transcription activator‐like (TAL) effector from Xanthomonas citri ssp. citri, triggers citrus canker resistance
Source: Mol Plant Pathol. 2019 Jul 5;20(10):1394–407. doi: 10.1111/mpp.12844 (PMC6792138; doi:10.1111/mpp.12844)
Supplement: Supplementary file 10 — Table S1. Bacterial strains. [file MPP-20-1394-s010.docx]

| **Strain** | **Relevant characteristics** | **Reference** |
| --- | --- | --- |
| **Xanthomonas citri** |  |  |
| X. citri A^T^ | Wild type | [Roeschlin](#_ENREF_40) *et al.,* (2017) |
| X. citri A^T^(pthA4) | Carries pBBR-*pthA4* | This study |
| X. citri 306 | Wild type | Da Silva *et al.,* (2002) |
| X. citri 306(pthA4^AT^) | Carries pBBR-*pthA4*^AT^ | This study |
| X. citri 306(pthA1^AT^) | Carries pBBR-*pthA1*^AT^ | This study |
| X. citri 306(∆AD^AT^) | Carries pBBR-∆AD^AT^ | This study |
| X. citri T | Wild type | [Roeschlin](#_ENREF_40) *et al.,* (2017) |
| X. citri T(pthA4^AT^) | Carries pBBR-*pthA4*^AT^ | This study |
| X. citri T(pthA1^AT^) | Carries pBBR-*pthA1*^AT^ | This study |
| ∆pthA4 | *X. citri* 306 *pthA4* deletion mutant | Soprano *et al.,* (2013) |
| ∆pthA1 | *X. citri* 306 *pthA1* deletion mutant | Yukari Abe & Benedetti (2015) |
| ∆pthA1,4 | *X. citri* 306 *pthA1,4* deletion mutant | Yukari Abe & Benedetti (2015) |
| ∆pthA4:pthA1^AT^ | Complement with pBBR-*pthA1*^AT^ | This study |
| ∆pthA4:pthA4^AT^ | Complement with pBBR-*pthA4*^AT^ | This study |
| ∆pthA4:∆NLS^AT^ | Complement with pBBR-∆NLS^AT^ | This study |
| ∆pthA4:mutNLS^AT^ | Complement with pBBR-mutNLS^AT^ | This study |
| ∆pthA4:SV40^AT^ | Complement with pBBR-SV40^AT^ | This study |
| ∆pthA4:∆AD^AT^ | Complement with pBBR-∆AD^AT^ | This study |
| ∆pthA4:∆AD | Complement with pBBR-∆AD | This study |
| **Agrobacterium tumefasciens** |  |  |
| GV3101 | C58C1 | Van Larebeke *et al.,* (1974) |
| 35S::pthA4^AT^ | GV3101 carries pCHF3*-pthA4*^AT^ | This study |
| 35S::pthA4 | GV3101 carries pCHF3-*pthA4* | This study |
| 35S::∆NLS^AT^ | GV3101 carries pCHF3-∆NLS^AT^ | This study |
| 35S::mutNLS^AT^ | GV3101 carries pCHF3- mutNLS^AT^ | This study |
| 35S::∆NLS^AT^-SV40 | GV3101 carries pCHF3-∆NLS^AT^-SV40 | This study |
| 35S::∆AD^AT^ | GV3101 carries pCHF3-∆AD^AT^ | This study |
| **Escherichia coli** |  |  |
| E. coli DH5α | *hsdR recA lacZYA ϕ*80 *lac*Z∆M15 | ThermoFisher Scientific, Waltham, MA, USA |
| E. coli BL-21 | fhuA2 [lon] ompT gal (λ DE3) [dcm] ∆hsdS λ DE3 = λ sBamHIo ∆EcoRI-B | New England Biolabs, Inc., Massachusetts, USA |

**Table S1.** Bacterial strains.
