# Supplementary material for: PthA4AT, a 7.5‐repeats transcription activator‐like (TAL) effector from Xanthomonas citri ssp. citri, triggers citrus canker resistance
Source: Mol Plant Pathol. 2019 Jul 5;20(10):1394–407. doi: 10.1111/mpp.12844 (PMC6792138; doi:10.1111/mpp.12844)
Supplement: Supplementary file 11 — Table S2. Plasmids used in this study. [file MPP-20-1394-s011.docx]

**Table S2.** Plasmids used in this study.

| **Plasmids** | **Relevant genotype^a^** | **Reference** |
| --- | --- | --- |
| pUC18 | *lacZ,* Ap^R^ | Invitrogen |
| pUC57-RR | pUC57 containing the *Bam*HI external fragments of *pthA* fused at 5' with *pthA4* native promoter and 3' with 3xFLAG tag, Ap^R^ | This study, Text S1 |
| pMAL-c2 | Maltose-binding protein fusion, Ap^R^ | New England Biolabs |
| pBBR | pBBR1-MCS2 derivative, mob-site, LacZα+; Km^R^ | Yaryura *et al.,* 2014 |
| pBBR-pthA4 | pBBR containing *pthA4* gene ^b^ of *X. citri* 306 | This study |
| pBBR-pthA4^AT^ | pBBR containing *pthA4* gene ^b^ of *X. citri* A^T^ | This study |
| pBBR-pthA1^AT^ | pBBR containing *pthA1* gene ^b^ of *X. citri* A^T^ | This study |
| pBBR-∆NLS^AT^ | pBBR containing *pthA4*^AT^ gene ^b^ with a deletion of 83 aminoacids on NLS domain | This study |
| pBBR-mutNLS^AT^ | pBBR containing *pthA4*^AT^ gene ^b^ with point mutations on NLS domain | This study |
| pBBR-SV40^AT^ | pBBR containing ∆NLS^AT^ gene ^b^ with an insertion of SV40 signal on NLS domain | This study |
| pBBR-∆AD^AT^ | pBBR containing *pthA4*^AT^ gene ^b^ with a deletion of 27 aminoacids on AD domain | This study |
| pCHF3 | Binary vector with 35S CaMV promoter; Spc^R^ | Jarvis *et al.,* 1998 |
| 35S::pthA4^AT^ | pCHF3 containing 35S fused with *pthA4^AT^* gene | This study |
| 35S::pthA4 | pCHF3 containing 35S fused with *pthA4* gene | This study |
| 35S::∆NLS^AT^ | pCHF3 containing *pthA4*^AT^ gene ^b^ with a deletion of 83 aminoacids on NLS domain | This study |
| 35S::mutNLS^AT^ | pCHF3 containing *pthA4*^AT^ gene ^b^ with point mutations on NLS domain | This study |
| 35S::∆NLS^AT^-SV40 | pCHF3 containing ∆NLS^AT^ gene ^b^ with an insertion of SV40 signal on NLS domain | This study |
| 35S::∆AD^AT^ | pCHF3 containing *pthA4*^AT^ gene ^b^ with a deletion of 27 aminoacids on AD domain | This study |
| pCAMBIA1303 | Binary vector with 35SCaMV promoter and gusA:mgfp5 reporter genes; Km^R^ | CAMBIA, Canberra, Australia |

^a^ Ap^R^, ampicillin resistance; Km^R^, kanamycin resistance; NLS, nuclear localization signal; AD, activation domain.

^b^ All *pthA*s are fused at 5´ with *pthA4* native promoter and 3´ with 3xFLAG tag
