# Supplementary material for: PthA4AT, a 7.5‐repeats transcription activator‐like (TAL) effector from Xanthomonas citri ssp. citri, triggers citrus canker resistance
Source: Mol Plant Pathol. 2019 Jul 5;20(10):1394–407. doi: 10.1111/mpp.12844 (PMC6792138; doi:10.1111/mpp.12844)
Supplement: Supplementary file 14 — Table S5. List of oligonucleotide primers used in this study. [file MPP-20-1394-s014.docx]

**Table S5.** List of oligonucleotide primers used in this study.

| **Primer name** | **Sequence (5´🡪 3´)** | **Application** |
| --- | --- | --- |
| Cs1 (fw)  Cs1 (rv) | CCGGGATCCTTGACCAACTTGACAACCATTTT  CCGCCATGGATGGCCGTTTTCCTCTCTG | [Amplification](#_ENREF_164) of orange1.1g019568 promoter |
| Cs2 (fw)  Cs2 (rv) | CCGTCTAGATATCATGCTTGTTGCTTCTGC  CCGCCATGGTGATTCCCAAAATTGTTAAATGC | [Amplification](#_ENREF_164) of orange1.1g047725 promoter |
| Cs3 (fw) | CCGTCTAGATTGTTCGCATTAATTGAAGGAC | [Amplification](#_ENREF_164) of orange1.1g038742 promoter |
| Cs3 (rv) | CCGCCATGGCGTCACATGAGAATGAGACTGAG |  |
| Cs4 (fw) | CCGTCTAGATCATACAACCTTTGAATTTTCAGC | [Amplification](#_ENREF_164) of orange1.1g048430 promoter |
| Cs4 (rv) | CACACCCAACTTAGCCATGA |  |
| Cs5 (fw)  Cs5 (rv) | CCGTCTAGATATTCCCTTCTTCTTCTCTTTTGG  CCGCCATGGTTTCTAATTTCGCCGTTGCT | [Amplification](#_ENREF_164) of orange1.1g048684 promoter |
| mEBE (rw) | CCGGAATTCATGTCGAAATATGGTTAAGTGGAA | Internal primer for Cs4 EBE mutation |
| mEBE (fw) | CCGGAATTCTTCTTCTTTCCGTACGTTATCAACAAT | Internal primer for Cs4 EBE mutation |
|  |  |  |

PCRs were performed for 30 cycles according to the following conditions: denaturation at 95°C for 5 min, annealing at 60°C for 30 s and extension at 72°C for 1 min 30 s. Underlined sequences are restriction enzymes *Bam*HI, *Xba*I and *Nco*I. Red letters show Cs4 EBE mutation.
