# Supplementary material for: PthA4AT, a 7.5‐repeats transcription activator‐like (TAL) effector from Xanthomonas citri ssp. citri, triggers citrus canker resistance
Source: Mol Plant Pathol. 2019 Jul 5;20(10):1394–407. doi: 10.1111/mpp.12844 (PMC6792138; doi:10.1111/mpp.12844)
Supplement: Supplementary file 15 — Table S6. List of motifs variants obtained with PBM11 protein‐binding microarrays for PthA4AT. [file MPP-20-1394-s015.docx]

**Table S6.** List of motifs variants obtained with PBM11 protein-binding microarrays for PthA4^AT^.

| **9-mer** | **9-mer** | **E-score** | **Median** | **Z-score** |
| --- | --- | --- | --- | --- |
| AAGGTAATA | TATTACCTT | 0.48119 | 429817.57 | 9.2908 |
| AGGTAATAA | TTATTACCT | 0.47635 | 427323.20 | 8.8393 |
| AAGGTGATA | TATCACCTT | 0.47606 | 429783.77 | 9.2847 |
| GAGGTAATA | TATTACCTC | 0.46630 | 419827.74 | 7.4667 |
| AGGTGATAA | TTATCACCT | 0.46432 | 416898.64 | 6.9236 |
| AAGGTTATA | TATAACCTT | 0.46180 | 417644.11 | 7.0622 |
| AGGTGATAT | ATATCACCT | 0.45985 | 418419.41 | 7.2060 |
| GGGTAATAA | TTATTACCC | 0.45967 | 417089.98 | 6.9592 |
| GGGGTAATA | TATTACCCC | 0.45830 | 417152.31 | 6.9708 |
| ATTATTACC | GGTAATAAT | 0.45638 | 417152.31 | 6.9708 |
| AAGGCGATA | TATCGCCTT | 0.45391 | 414256.90 | 6.4305 |
| AGGCGATAT | ATATCGCCT | 0.45041 | 415054.87 | 6.5798 |
