# Supplementary material for: PthA4AT, a 7.5‐repeats transcription activator‐like (TAL) effector from Xanthomonas citri ssp. citri, triggers citrus canker resistance
Source: Mol Plant Pathol. 2019 Jul 5;20(10):1394–407. doi: 10.1111/mpp.12844 (PMC6792138; doi:10.1111/mpp.12844)
Supplement: Supplementary file 16 — Data S1. Design sequences for synthesis and cloning of the full‐length of pthAs and mutant construction. [file MPP-20-1394-s016.docx]

**Data S1**. **Design sequences for synthesis and cloning of the full-length of *pthAs* and mutant construction**

Synthesized sequence on pUC57 plasmid. Grey background indicates the predicted sequence for *pthA4* promoter from *X. citri* 306. Blue letters show rbs sequence. Restriction enzymes *Kpn*I, *Xho*I, *Xba*I, *Bam*HI, *Bgl*II, *Sma*I and *Hind*III are highlighted in red. The predicted translation start is underlined. The 3´ conserved sequence of *pthAs* is highlighted with yellow background and the 3xflag tag is mark with black bold letters.

>RR design sequence

**GGTACCCTCGAG**CTATCGCGGCGAGGCCGCCAGCGCACATAACGCGAGATTCCACGCACTGGCGCGGTGCCTCCGTGGCCCAGCAGGGCGGTCGGGCGGTCGCATGACGGGGTTTATGCATTCTTGCATCGCCCGGGCGCAACCGCTATTGTGCACATGCACCACCGACCGGACCCTGGAGACAGGTGTGGTGCCTCGATAGATCGATAGCTTCACCCGATCTATCCCTTGCCTGGAGCGGGATACGGCAGAAATGCCCAAAGAGCCTACCGAGTAGTGAATACCGAAAGCGCTCCAGCGATGGGGCGCTTTCTTTTTATAGGGCAATGGTCTGTTCGGCCAAGCGCCGCCGGTACACAGTCTCAACCTTGTAGAGCTTCCCGCGCGGGAGTTTATGGATGGTTGTCCCGTCGACGGGGTAGCAGGAGATGAGGGTCGGCAGGGATTGGTGTAAAAAACAGCCAAAAGTGAGCTAACTCGCTGTCAGCACAGAAATTTTTCACAACCTTCTGCCGATCCTCCATGCGGGTCCGTGATCGCCTTCATGTCTGCGCCTCACCCTGGTCGTCGAGGGTTGCCAGGATCACCCGAAGTTGTGTACTGCCATGCGGCCTCGGAAGCTATGTAGGAACCACAGACCGCTAGTCTGGAGGCGACCATGTAAAGAGGTA**TCTAGA**AT**GGATCC**TGGTACGCCCACGGCTGCCGACCTGGCAGCGTCCAGCACCGTGATGCGGGAACAAGATGAGGACCCCTTCGCAGGGGCAGCGGATGATTTCCCGGCATTCAACGAAGAGGAGCTCGCATGGTTGATGGAGCTATTGCCT**AGATCTGATTACAAAGATCATGATGGTGACTATAAGGACCACGACATCGATTACAAAGATGATGATGATTGACCCGGG**CCCGTCGACTGCAGAGGCCTGCATGC**AAGCTT**

Synthesized sequence for NLS deletion (∆NLS). Restriction enzymes BclI and *Hind*III are highlighted in red. Bars indicate the deletion site.

**>**∆NLS

**tgatca**aaagaaccaatcgccgtattcccgaacgcacatcccatcgcgttgccgaccacgcgcaagtggttcgcgtgctgggttttttccagtgccactcccacccagcgcaagcatttgatgacgccatgacgcagttcgggatgagcaggcacgggttgttacagctctttcgcagagtgggcgtcaccgaactcgaagcccgcagtggaacgctccccccagcctcgcagcgttgggaccgtatcctccaggcatcagggatg**//**accagtatcgggggcggcctcccggatcctggtacgcccacggctgccgacctggcagcgtccagcaccgtgatgcgggaacaagatgaggaccccttcgcaggggcagcggatgatttcccggcattcaacgaagaggagctcgcatggttgatggagctattgcctagatctGATTACAAAGATCATGATGGTGACTATAAGGACCACGACATCGATTACAAAGATGATGATGATTGACCCGGGCCCGTCGACTGCAGAGGCCTGCATGC**AAGCTT**

Synthesized sequence for SV40 insertion (SV40). Restriction enzymes *Bcl*I and *Hind*III are highlighted in red. Green background indicates SV40 insertion.

>SV40

**tgatca**aaagaaccaatcgccgtattcccgaacgcacatcccatcgcgttgccgaccacgcgcaagtggttcgcgtgctgggttttttccagtgccactcccacccagcgcaagcatttgatgacgccatgacgcagttcgggatgagcaggcacgggttgttacagctctttcgcagagtgggcgtcaccgaactcgaagcccgcagtggaacgctccccccagcctcgcagcgttgggaccgtatcctccaggcatcagggatgCGGCCGAAGAAGAAGCGCAAGGTCTCGaccagtatcgggggcggcctcccggatcctggtacgcccacggctgccgacctggcagcgtccagcaccgtgatgcgggaacaagatgaggaccccttcgcaggggcagcggatgatttcccggcattcaacgaagaggagctcgcatggttgatggagctattgcctagatctgattacaaagatcatgatggtgactataaggaccacgacatcgattacaaagatgatgatgattgacccgggcccgtcgactgcagaggcctgcatgc**aagctt**

Synthesized sequence for NLS mutations (mutNLS). Restriction enzymes *Bcl*I and *Hind*III are highlighted in red. Green background indicates NLS mutations.

>mutNLS

**tgatca**aaagaaccaatcgccgtattcccgaacgcacatcccatcgcgttgccgaccacgcgcaagtggttcgcgtgctgggttttttccagtgccactcccacccagcgcaagcatttgatgacgccatgacgcagttcgggatgagcaggcacgggttgttacagctctttcgcagagtgggcgtcaccgaactcgaagcccgcagtggaacgctccccccagcctcgcagcgttgggaccgtatcctccaggcatcagggatgacaaaggacacaccgtcccctacttcaactcaaacgccggaccaggcgtctttgcatgcattcgccgattcgctggagcgtgaccttgatgcgcccagcccaacgcacgagggagatcagaggcgggcaagcagccgtacaggggccggatcggatcgtgctgtcaccggtccctccgcacagcaatcgttcgaggtgcgcgttcccgaacagcgcgatgcgctgcatttgcccctcagttggagggtaacacacgcgcttaccagtatcgggggcggcctcccggatcctggtacgcccacggctgccgacctggcagcgtccagcaccgtgatgcgggaacaagatgaggaccccttcgcaggggcagcggatgatttcccggcattcaacgaagaggagctcgcatggttgatggagctattgcctagatctgattacaaagatcatgatggtgactataaggaccacgacatcgattacaaagatgatgatgattgacccgggcccgtcgactgcagaggcctgcatgc**aagctt**

Synthesized sequence for AD deletion (∆AD). Restriction enzymes *Bcl*I and *Hind*III are highlighted in red. Bars indicate the deletion site.

>∆AD

**TGATCA**AAAGAACCAATCGCCGTATTCCCGAACGCACATCCCATCGCGTTGCCGACCACGCGCAAGTGGTTCGCGTGCTGGGTTTTTTCCAGTGCCACTCCCACCCAGCGCAAGCATTTGATGACGCCATGACGCAGTTCGGGATGAGCAGGCACGGGTTGTTACAGCTCTTTCGCAGAGTGGGCGTCACCGAACTCGAAGCCCGCAGTGGAACGCTCCCCCCAGCCTCGCAGCGTTGGGACCGTATCCTCCAGGCATCAGGGATGAAAAGGGCCAAACCGTCCCCTACTTCAACTCAAACGCCGGACCAGGCGTCTTTGCATGCATTCGCCGATTCGCTGGAGCGTGACCTTGATGCGCCCAGCCCAACGCACGAGGGAGATCAGAGGCGGGCAAGCAGCCGTAAACGGTCCCGATCGGATCGTGCTGTCACCGGTCCCTCCGCACAGCAATCGTTCGAGGTGCGCGTTCCCGAACAGCGCGATGCGCTGCATTTGCCCCTCAGTTGGAGGGTAAAACGCCCGCGTACCAGTATCGGGGGCGGCCTCCCGGATCCTGGTACGCCCACGGCTGCCGACCTGGCAGCGTCCAGCACCGTGATGCGG**//**CTATTGCCTAGATCTGATTACAAAGATCATGATGGTGACTATAAGGACCACGACATCGATTACAAAGATGATGATGATTGACCCGGGCCCGTCGACTGCAGAGGCCTGCATGC**AAGCTT**
